# A systematic review of the diagnostic performance of orthopedic physical examination tests of the hip

**DOI:** 10.1186/1471-2474-14-257

**Published:** 2013-08-30

**Authors:** Labib Ataur Rahman, Sam Adie, Justine Maree Naylor, Rajat Mittal, Sarah So, Ian Andrew Harris

**Affiliations:** 1 South West Sydney Clinical School, University of New South Wales, P.O. Box 906, Caringbah, NSW 2229, Australia; 2 Orthopaedic Department, Liverpool Hospital, P.O. Box 906, Caringbah, NSW 2229, Australia; 3 Whitlam Orthopaedic Research Centre, P.O. Box 906, Caringbah, NSW 2229, Australia

**Keywords:** Physical examination/physical tests, Hip/Hip joint, Diagnosis, Sensitivity and specificity, Predictive values, Likelihood ratios, Systematic review, Orthopedics

## Abstract

**Background:**

Previous reviews of the diagnostic performances of physical tests of the hip in orthopedics have drawn limited conclusions because of the low to moderate quality of primary studies published in the literature. This systematic review aims to build on these reviews by assessing a broad range of hip pathologies, and employing a more selective approach to the inclusion of studies in order to accurately gauge diagnostic performance for the purposes of making recommendations for clinical practice and future research. It specifically identifies tests which demonstrate strong and moderate diagnostic performance.

**Methods:**

A systematic search of Medline, Embase, Embase Classic and CINAHL was conducted to identify studies of hip tests. Our selection criteria included an analysis of internal and external validity. We reported diagnostic performance in terms of sensitivity, specificity, predictive values and likelihood ratios. Likelihood ratios were used to identify tests with strong and moderate diagnostic utility.

**Results:**

Only a small proportion of tests reported in the literature have been assessed in methodologically valid primary studies. 16 studies were included in our review, producing 56 independent test-pathology combinations. Two tests demonstrated strong clinical utility, the patellar-pubic percussion test for excluding radiologically occult hip fractures (negative LR 0.05, 95% Confidence Interval [CI] 0.03-0.08) and the hip abduction sign for diagnosing sarcoglycanopathies in patients with known muscular dystrophies (positive LR 34.29, 95% CI 10.97-122.30). Fifteen tests demonstrated moderate diagnostic utility for diagnosing and/or excluding hip fractures, symptomatic osteoarthritis and loosening of components post-total hip arthroplasty.

**Conclusions:**

We have identified a number of tests demonstrating strong and moderate diagnostic performance. These findings must be viewed with caution as there are concerns over the methodological quality of the primary studies from which we have extracted our data. Future studies should recruit larger, representative populations and allow for the construction of complete 2×2 contingency tables.

## Background

The diagnostic value of many physical tests in orthopedic practice has been called into question and a number of these tests have been found to correspond poorly with anatomical models [1,2]. In some cases, clinicians proceed directly to more invasive or technologically-involved ‘definitive’ investigations, however this is not always desirable, practical or economical [3]. For example, the more direct approach has been blamed for diagnostic delays and misclassification of hip joint pathologies [4].

Recently, several diagnostic reviews of physical tests of the hip have been published [5-8] and they generally support the view that most studies are of low to moderate quality. Three of these reviews examined labral pathologies and/or femoroacetabular impingement [5,6,8] while a fourth looked at a wider range of pathologies [7]. This systematic review aims to build on these reviews by assessing a broad range of hip pathologies, and employing a more selective approach to the inclusion of studies in order to accurately gauge diagnostic performance for the purposes of making recommendations for clinical practice and future research. We aim to determine:

i) which physical tests of the hip or physical clinical prediction rules have valid evidence from which their diagnostic performance in clinical practice can be calculated; and

ii) whether any physical tests or clinical prediction rules have strong diagnostic utility; and

iii) whether any physical tests or clinical prediction rules have moderate diagnostic utility.

## Methods

In this systematic review, a preliminary search of various textbooks, medical journal databases, websites and grey literature sources was conducted to identify physical tests of the hip. Subsequently, an electronic database search strategy was developed, aided by a medical librarian (see Additional file 1), and applied to Medline (1950-July 2010), Embase (1980-July 2010), Embase Classic (1947–1979) and the Cumulative Index to Nursing and Allied Health Literature (CINAHL) (1982-July 2010). A follow up search was performed in March 2013 using Medline, Embase and CINAHL to identify studies published in the interim period following the original search (see Additional file 1).

Studies included in our review were required to:

i) compare a physical (index) test for the diagnosis of a particular hip pathology against a ‘gold standard’ (reference) test representing the true diagnostic result. Physical tests were defined as non-invasive bedside maneuvers, beyond inspection, point tenderness and palpation alone, which were intended to increase the probability of a particular diagnosis; and

ii) report sufficient information to construct complete 2×2 contingency tables; and

iii) recruit predominantly adult populations (where ages were indicated); and

iv) be written in English.

Studies were excluded if they:

i) used physical tests under anesthesia or intra-operatively; or

ii) used physical tests to diagnose vascular or neurologic pathologies.

Studies were also excluded if they did not meet our criteria for internally and externally valid methodology. These criteria are listed below.

iii) For the purposes of internal validity, reference tests could not: (1) be dependent upon the index test result for interpretation, (2) be discredited for diagnosing the chosen pathology, or (3) allow for only partial construction of 2×2 contingency tables (e.g. by excluding persons with negative index test results from the study).

iv) For the purposes of external validity, (1) the sample population had to reasonably represent a typical population presenting for diagnosis in clinical practice (e.g. they could not use healthy or asymptomatic controls who had no indications for testing), and (2) the index test needed to provide a threshold for dichotomizing results.

Assessments of validity were made independently by two authors and disputes arbitrated by a third author. No further restrictions were placed on study design, date of publication or clinical setting.

For the literature search in 2010, one author screened citations for inclusion on the basis of their title. The remaining citations were assessed independently by two authors, first by title and abstract and then by full text. Opposing views regarding inclusion were resolved by arbitration with the remaining authors. When new tests were identified, new search strategies were executed for them using Medline, Embase and Embase Classic (see Additional file 1). The follow up literature search and sorting process in March 2013 were conducted entirely by a single author.

The diagnostic performances of included physical tests are presented in terms of sensitivity, specificity, predictive values and likelihood ratios (LRs) with the latter being used to further identify tests demonstrating “strong” and “moderate” diagnostic utility. We favor the use of likelihood ratios because they offer the most valuable and comprehensive diagnostic information in the individual patient [9,10]. Roughly speaking, tests with positive LRs greater than or equal to 10 or negative LRs less than or equal to 0.1 will cause almost conclusive, “strong” changes in post-test probability of disease. Positive LRs between 5 and 9.99 and negative LRs between 0.11 and 0.2 cause “moderate” changes in post-test probability [9]. In order to limit the uncertainty caused by studies recruiting small sample populations, we required “strong” tests to meet our likelihood ratio criteria within their entire 95% confidence intervals (otherwise the test was classified as “moderate”). When diagnostic data was only presented in the form of percentages or fractions, we attempted to revert it back to integer form to determine the original population numbers in each diagnostic category of a 2x2 contingency table. We only pooled data from studies involving the exact same index test and target pathology.

## Results

**Figure 1:**
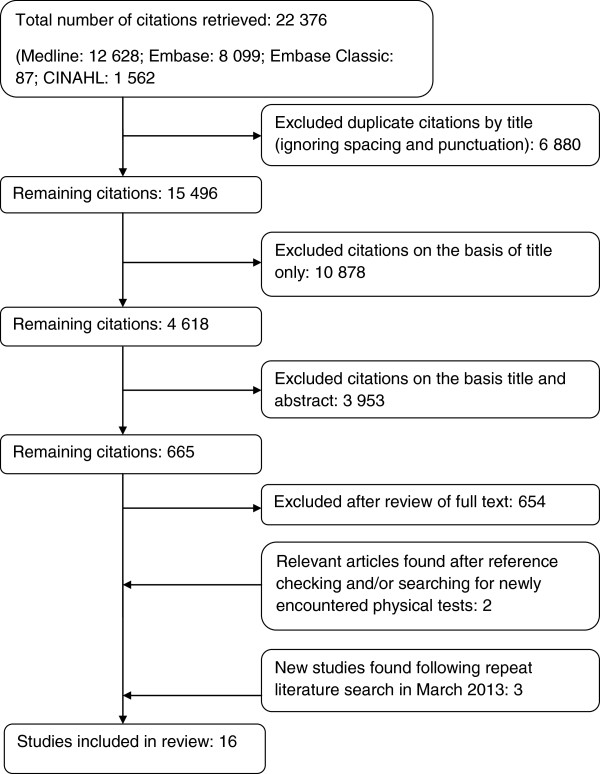
Flow diagram of study inclusions and exclusions.

Only a small proportion of hip tests identified in our preliminary search had their diagnostic performance assessed in methodologically valid primary studies. We identified sixteen studies containing data that satisfied our inclusion and exclusion criteria [11-26] (Figure 1). This produced a total of 56 independent test-pathology combinations (Additional file 2).

Two physical tests demonstrated strong diagnostic utility with the patellar-pubic percussion (PPP) test strongly excluding radiologically occult hip fractures (negative LR 0.05, 95% CI 0.03-0.08) [26], and the hip abduction sign strongly diagnosing sarcoglycanopathies in patients with known muscular dystrophies (positive LR 34.29, 95% CI 10.97-122.30) [20] (Table 1). The original description of these tests from the primary studies can be found in Additional file 2.

**Table 1:** **Diagnostic performances of independent physical test-hip pathology combinations with strong clinical diagnostic utility**^**a**^

**Study**	**Test**	**Pathology**	**Reference standard**	**Sensitivity**	**Specificity**	**PPV**	**NPV**	**+LR**	**-LR**
				**(95% CI)**	**(95% CI)**			**(95% CI)**	**(95% CI)**
				**TP/ (TP+FN)**	**TN/ (TN+FP)**				
**Khadilkar et al. 2001 **[20]^**b**^	Hip Abduction Sign	Sarcoglycan opathies in patients with known muscular dystrophy	Immunocyto chemistry	0.76	0.98	0.89	0.95	34.29	0.24
				0.61-0.83	0.94-0.99			10.97 – 122.30	0.17-0.41
				16/21	88/90				
**Tiru et al. 2002 **[26]	Patellar-Pubic Percussion Test	Traumatic Fracture (Radiologically Occult)	Repeat Radiography, Bone Scintigraphy, MRI or CT	0.96	0.86	0.98	0.75	6.73	0.05
				0.94-0.97	0.74-0.93			3.61-14.00	0.03-0.08
				245/ 255	30/35				

Positive Predictive Value (PPV), Negative Predictive Value (NPV), Positive Likelihood Ratio (+LR), Negative Likelihood Ratio (−LR), 95% Confidence Interval (95% CI), True Positives (TP), False Positives (FP), True Negatives (TN), False Negatives (FN). All values rounded to 2 decimal places.

^a^Strong diagnostic utility defined as either +LR ≥ 10 or -LR ≤ 0.1 where entire 95% confidence interval satisfies these thresholds. Moderate diagnostic utility defined as +LR > 5 or -LR < 0.2 without satisfying the criteria for strong diagnostic utility.

^b^10 healthy controls that tested negative with the index test were removed from our calculations.

Fifteen independent test-pathology combinations demonstrated, at most, moderate diagnostic utility (Table 2). These included five tests for diagnosing symptomatic osteoarthritis [25], seven tests for diagnosing loosening of various components post-total hip arthroplasty [23] and three tests for diagnosing and excluding various hip fractures [11,13,24].

**Table 2:** **Diagnostic performances of independent physical test-hip pathology combinations with moderate clinical diagnostic utility**^**a**^

**Study**	**Test**	**Pathology**	**Reference standard**	**Sensitivity**	**Specificity**	**PPV**	**NPV**	**+LR**	**-LR**
				**(95% CI)**	**(95% CI)**			**(95% CI)**	**(95% CI)**
				**TP/ (TP+FN)**	**TN/ (TN+FP)**				
**Symptomatic Osteoarthritis:**									
**Sutlive et al. 2008 **[25]	Pain on Abduction and/or Adduction. Patient Supine.	Symptomatic Osteoarthritis	Radiography	0.33	0.94	0.70	0.77	5.67	0.71
				0.20-0.42	0.89-0.98			1.76-19.05	0.59-0.90
				7/21	48/51				
**Sutlive et al. 2008 **[25]	Squat Test	Symptomatic Osteoarthritis	Radiography	0.24	0.96	0.71	0.75	6.07	0.79
				0.13-0.31	0.91-0.99			1.46-26.32	0.70-0.96
				5/21	49/51				
**Sutlive et al. 2008 **[25]	5-Part Clinical Prediction Rule^b^ (≥3 Variables Positive)	Symptomatic Osteoarthritis	Radiography	0.71	0.86	0.68	0.88	5.20	0.33
				0.55-0.84	0.79-0.91			2.66-9.57	0.18-0.57
				15/21	44/51				
**Sutlive et al. 2008 **[25]	5-Part Clinical Prediction Rule^b^ (≥4 Variables Positive)	Symptomatic Osteoarthritis	Radiography	0.48	0.98	0.91	0.82	24.29	0.53
				0.34-0.52	0.93-1.00			4.64-145.01	0.49-0.71
				10/21	50/51				
**Sutlive et al. 2008 **[25]	5-Part Clinical Prediction Rule^b^ (All 5 Variables Positive)	Symptomatic Osteoarthritis	Radiography	0.14	0.98	0.75	0.74	7.29	0.87
				0.06-0.18	0.95-1.00			1.09-50.33	0.82-1.00
				3/21	50/51				
**Loosening of Components Post-Total Hip Arthroplasty (THA)**									
**Pooled Data: Röder et al. 2003 **[23]	Pain on Axial Compression	Uncemented Acetabular Cup Loosening Post-THA	Radiography	0.08	0.99	0.20	0.98	12.15	0.93
				0.03-0.17	0.99-1.00			4.33 – 32.83	0.84 – 0.97
				4/49	2365/ 2381				
**Pooled Data: Röder et al. 2003 **[23]	Pain on Internal Rotation	Uncemented Acetabular Cup Loosening Post-THA	Radiography	0.20	0.97	0.12	0.07	6.09	0.83
				0.12 – 0.31	0.97 – 0.97			3.39 – 10.37	0.71 – 0.91
				11/ 55	2297/ 2375				
**Pooled Data: Röder et al. 2003 **[23]	Pain on External Rotation	Uncemented Acetabular Cup Loosening Post-THA	Radiography	0.06	0.99	0.14	0.98	7.67	0.95
				0.02 – 0.14	0.99 – 0.99			2.45 – 22.97	0.86 – 0.99
				3/ 49	2362/ 2381				
**Pooled Data: Röder et al. 2003 **[23]	Pain on External Rotation	Cemented Acetabular Cup Loosening Post-THA	Radiography	0.02	1.00	0.25	0.95	5.96	0.99
				0.00 – 0.04	1.00 – 1.00			0.86 – 41.13	0.96 – 1.00
				1/67	1194 /1197				
**Pooled Data: Röder et al. 2003 **[23]	Pain on Axial Compression Pain on Axial Compression	Uncemented Femoral Stem Loosening Post-THA Uncemented Femoral Stem Loosening Post-THA	Radiography	0.07	0.99	0.25	0.96	6.61	0.95
			Radiography	0.02 – 0.15	0.99 – 1.00	0.25	0.96	1.55 – 27.35 6.61	0.86 – 0.99
				0.07	0.99				0.95
**Pooled Data: Röder et al. 2003 **[23]	Pain on External Rotation Pain on External Rotation	Cemented Femoral Stem Loosening Post-THA Cemented Femoral Stem Loosening Post-THA	Radiography	0.03	1.00	0.41	0.22	8.91	0.97
			Radiography	0.02 – 0.05	1.00 – 1.00	0.41	0.22	3.53 – 22.43	0.95 – 0.99
				0.03	1.00				0.97
								8.91	
**Pooled Data: Röder et al. 2003 **[23]	Flexion ROM < 70°	Uncemented Femoral Stem Loosening	Radiography	0.15	0.98	0.25	0.95	5.97	0.87
				0.06-0.28	0.97-0.98			1.95-16.128	0.73-0.97
				5/34	594/609				
**Hip Fractures:**									
**Adams et al. 1997 **[11]	Patellar-Pubic Percussion	Traumatic Fracture	Radiography	0.79	0.95	0.94	0.84	17.37	0.22
				0.65-0.83	0.84-0.99			3.97-98.43	0.17-0.42
				15/19	21/22				
**Bache et al. 1984 **[13]	Bartford test	Fractured neck of femur	Radiography	0.91	0.82	0.86	0.88	5.01	0.11
				083 – 0.96	0.72 – 0.88			2.92 – 8.20	0.04 – 0.28
				51/56	36/44				
**Shin et al. 1996 **[24]	Pain on Log Roll Test	Femoral Neck Stress Fracture (radiologically occult but suggestive bone scintigraphy)	6-week Follow up Radiography	1.00	0.33	0.76	1.00	1.50^a^	0.10
				0.90-1.00	0.12-0.33			1.00 – 1.72^a^	0.01 – 0.98^a^
				13/13	2/6				

Positive Predictive Value (PPV), Negative Predictive Value (NPV), Positive Likelihood Ratio (+LR), Negative Likelihood Ratio (−LR), 95% Confidence Interval (95% CI), True Positives (TP), False Positives (FP), True Negatives (TN), False Negatives (FN), Range of Motion (ROM). All values rounded to 2 decimal places. When one of the cells of the 2×2 contingency table contained the value ‘zero’, we added 0.5 to each cell in order to calculate likelihood ratio values and their confidence intervals.

^a^Strong diagnostic utility defined as either +LR ≥ 10 or -LR ≤ 0.1 where entire 95% confidence interval satisfies these thresholds. Moderate diagnostic utility defined as +LR > 5 or -LR < 0.2 without satisfying the criteria for strong diagnostic utility.

^b^Clinical Prediction Rule consisted of 5 variables: (1) self-reported squatting as an aggravating factor, (2) scour test with adduction causing groin or lateral pain, (3) active hip flexion causing late pain, (4) active hip extension causing hip pain, and (5) passive hip internal rotation less than or equal to 25°.

## Discussion

Previous reviews of physical tests have found much of the existing literature to be methodologically flawed and insufficient for guiding clinical practice. This review sought to identify clinically useful physical tests or combinations of tests that demonstrated strong and moderate diagnostic performance. This information could potentially be used to form future clinical prediction rules or guide future research. We found the PPP test strongly excluded radiologically occult hip fractures and the hip abduction sign strongly diagnosed sarcoglycanopathies in patients with known muscular dystrophies. In addition, we identified a number of tests with moderate usefulness for diagnosing and/or excluding hip fractures, symptomatic osteoarthritis and loosening of components post-THA.

While some of our results are promising at face value, the raw data needs to be considered in more detail.

Firstly, it is possible that we have overstated the utility of the PPP test since we have based our conclusions primarily on a single study by Tiru et al. [26]. Two other studies recruiting smaller populations [11,13] also employed the principle of osteophony when testing for hip fractures and found only moderate diagnostic utility. We did not pool the data from these studies they tested for radiologically apparent fractures, and the Bartford test employed by Bache and Cross [13] auscultated for sound transmitted by a tuning fork rather than percussion.

The hip abduction sign may also not perform as strongly as we suggested because Khadilkar and Singh [20] relied on retrospective testing of patients with known diagnoses of variable duration and severity. It is therefore possible that some of the recruited sample population may not have reflected clinical practice. Khadilkar and Singh’s [20] findings need to be confirmed prospectively in a pre-diagnosis setting.

There was significant uncertainty about the true diagnostic performance of some of the moderately useful physical tests because of the small sample populations recruited in the primary studies [11,13,24-26]. We suggest further testing with large sample populations would be of benefit to better assess if these tests should be considered for inclusion in future clinical prediction rules.

While we acknowledge that previous hip test reviews have found much of the literature to be methodologically flawed, we did not use cumulatively-scored quality assessment tools to analyze our data as the implications of these numerical values are not clear [27]. Instead, we used our methodological validity criteria to provide a minimum standard to serve our primary purpose, which was to identify tests with strong and moderate diagnostic performance for use in clinical practice. Although our criteria are generally consistent with quality assessment tools and have been empirically associated with design-related bias [28], we acknowledge that this does not eliminate all bias and that there remain significant shortcomings in the literature. We believe our criteria represent a reasonable compromise for the sake of drawing basic conclusions. That said, since our criteria have not been independently validated, we have reported data from excluded studies in Additional file 3 when complete 2×2 contingency tables could be formed and Additional file 4 for the remaining studies and case reports. There were some discrepancies between this review and those that have been previously published. In some instances this was explained by calculation errors and in others this was because we found there was insufficient information in the primary study to construct 2×2 contingency tables for calculation of diagnostic performance.

## Conclusions

There is valid evidence for the diagnostic performance of only a small proportion of physical tests of the hip in routine clinical practice. Two tests demonstrated strong diagnostic utility, the patellar-pubic percussion test for excluding radiologically occult hip fractures and the hip abduction sign for diagnosing sarcoglycanopathies in patients with known muscular dystrophies. In addition, we identified a number of tests with moderate usefulness for diagnosing and/or excluding hip fractures, symptomatic osteoarthritis and loosening of components post-THA. The primary studies from which our data are derived contain methodological flaws that bias their results. Future studies should recruit larger and more representative populations and allow for construction of complete 2×2 contingency tables.

## Abbreviations

THA: Total hip arthroplasty; +LR: Positive likelihood ratio; -LR: Negative likelihood ratio; CI: Confidence interval; CINAHL: Cumulative Index to Nursing and Allied Health Literature; FN: False negatives; FP: False positives; LR: Likelihood ratio; NPV: Negative predictive value; PPV: Positive predictive value; TN: True negatives; TP: True positives.

## Competing interests

The authors declare that they have no competing interests.

## Authors’ contributions

LAR contributed to the design of the review; acquisition, analysis and interpretation of data; and drafting and revising of the manuscript. SA contributed to the conception and design of the review; analysis and interpretation of data; and revising of the manuscript. JMN contributed to the conception and design of the review; analysis and interpretation of data; and drafting and revising of the manuscript. RM contributed to the conception and design of the review; analysis and interpretation of data; and revising of the manuscript. SS contributed to the acquisition, analysis and interpretation of data, and revising of the manuscript. IAH contributed to the conception and design of the study; analysis and interpretation of the data; and revision of the manuscript. All authors read and approved the final manuscript.

## Pre-publication history

The pre-publication history for this paper can be accessed here:

http://www.biomedcentral.com/1471-2474/14/257/prepub

## Supplementary Material

Additional file 1 **Search strategy for Medline, Embase, Embase Classic and CINAHL.** File shows search strategy, search terms and results for Medline, Embase, Embase Classic and CINAHL. Click here for file

Additional file 2 **Diagnostic performances of physical test-hip pathology combinations included in review.** File is a table of diagnostic characteristics of physical test-hip pathology combinations (sensitivity, specificity, positive and negative predictive values, and positive and negative likelihood ratios) from studies included in this review. Click here for file

Additional file 3 **Diagnostic performances of physical test-hip pathology combinations from excluded studies (2×2 contingency tables).** File is a table of diagnostic characteristics of physical test-hip pathology combinations (sensitivity, specificity, positive and negative predictive values, and positive and negative likelihood ratios) from excluded studies that allowed for the construction of complete 2×2 contingency tables. Click here for file

Additional file 4 **Overview of excluded studies and case reports not presented in complete 2×2 contingency tables.** File is a basic description of studies and case reports that were excluded from our studies and did not allow for the construction of complete 2×2 contingency tables (for example, because they excluded patients with negative index tests from their study). Click here for file
