# Supplementary material for: A systematic review of the diagnostic performance of orthopedic physical examination tests of the hip
Source: BMC Musculoskelet Disord. 2013 Aug 30;14:257. doi: 10.1186/1471-2474-14-257 (PMC3766647; doi:10.1186/1471-2474-14-257)
Supplement: Additional file 1 — Search strategy for Medline, Embase, Embase Classic and CINAHL. File shows search strategy, search terms and results for Medline, Embase, Embase Classic and CINAHL. [file 1471-2474-14-257-S1.docx]

**Additional file 1 - Search strategy for Medline, Embase, Embase Classic and CINAHL**

**Article title:** A systematic review of the diagnostic performance of orthopedic physical examination tests of the hip.

**Journal**: BMC Musculoskeletal Disorders

**Authors**: Labib A. Rahman^1^; Sam Adie^1,2,3^ ; Justine M. Naylor ^1,2,3^; Rajat Mittal^1,2,3^; Sarah So^1^; Ian A. Harris^1,2,3^

^1^South West Sydney Clinical School, University of New South Wales, ^2^Orthopaedic Department, Liverpool Hospital, ^3^Whitlam Orthopaedic Research Centre

**Explanatory Note**:

Our initial search strategy was composed of two parts.

Part I is the original search strategy. For the databases searched via Ovid (Medline, Embase and Embase Classic), our strategy can be understood as the combination of a ‘generic approach’ (Search 92) and a ‘specific approach’ (Search 97). The generic approach consisted of terms associated with physical examination of the hip using: (a) MeSH terms to identify articles that were properly indexed (Search 84), and (b) text words to identify articles that may be poorly indexed (Search 90). The specific approach combined named physical tests (Search 81) with: (a) MeSH terms for physical examination of the hip (Search 84), (b) text words for physical examination (Search 88), and (c) text words for hip joint anatomy (Search 89). For CINAHL, we employed a ‘generic’ strategy combining subject headings (S3) and basic search terms (S9).

Part II consist of specific, named physical tests that were identified during the review and searched on Medline, Embase and Embase Classic.

In order to keep our findings up to date, our search strategy was repeated again in 2013 with a few modifications (Part III). We repeated our basic search strategies for Medline and Embase, limiting our results from the year 2010 to March 3, 2013. In addition, we repeated our search strategy for CINAHL with limitations from June, 2010, to March 7, 2013.

**Part I: Original Search Strategy**

Medline (1950 to July 2010) via Ovid. This search was conducted on 20/07/2010.

| **No.** | **Search Terms** | **Results** |
| --- | --- | --- |
| **1** | actual leg length.tw. | 3 |
| **2** | allis$.tw. | 277 |
| **3** | galeazzi$.tw. | 151 |
| **4** | anvil$.tw. | 284 |
| **5** | heel strike.tw. | 416 |
| **6** | apparent leg length.tw. | 9 |
| **7** | Barlow$.tw. | 390 |
| **8** | berry$.tw. | 1408 |
| **9** | Bryant$.tw. | 341 |
| **10** | buttock sign.tw. | 1 |
| **11** | Chiene$.tw. | 5 |
| **12** | Craig$.tw. | 657 |
| **13** | Dial$.tw. | 97005 |
| **14** | ely$.tw. | 545 |
| **15** | heel to buttock.tw. | 4 |
| **16** | faber$.tw. | 213 |
| **17** | Patrick$.tw. | 497 |
| **18** | Flexion, Abduction External Rotation.tw. | 20 |
| **19** | Figure four.tw. | 7 |
| **20** | faddir.tw. | 0 |
| **21** | flexion adduction.mp. and internal rotation.tw. [mp=title, original title, abstract, name of substance word, subject heading word, unique identifier] | 25 |
| **22** | Fitzgerald$.tw. | 304 |
| **23** | Flexion Abduction.mp. and Internal Rotation.tw. [mp=title, original title, abstract, name of substance word, subject heading word, unique identifier] | 42 |
| **24** | Flexion Adduction Axial Compression.tw. | 0 |
| **25** | Flexion Internal Rotation Axial Compression.tw. | 0 |
| **26** | Freiberg$.tw. | 128 |
| **27** | Fulcrum$.tw. | 381 |
| **28** | Gauvain$.tw. | 0 |
| **29** | Hamstring 90.tw. | 0 |
| **30** | straight leg raising.tw. | 378 |
| **31** | Hanneqin$.tw. | 0 |
| **32** | Hibb$.tw. | 121 |
| **33** | hip abduction stress.tw. | 0 |
| **34** | hip adduction deformity.tw. | 1 |
| **35** | Scour$.tw. | 1078 |
| **36** | telescop$.tw. | 2363 |
| **37** | hop test.tw. | 128 |
| **38** | impingement test.tw. | 50 |
| **39** | Flexion Adduction Internal Rotation.tw. | 5 |
| **40** | impingement provocation.tw. | 1 |
| **41** | Jansen$.tw. | 230 |
| **42** | Laguerre$.tw. | 109 |
| **43** | Lasegue$.tw. | 146 |
| **44** | Lewin$.tw. | 333 |
| **45** | log roll.tw. | 20 |
| **46** | log roll test.tw. | 2 |
| **47** | Ludloff$.tw. | 57 |
| **48** | Lunge$.tw. | 193 |
| **49** | Malinger$.tw. | 1154 |
| **50** | McCarthy$.tw. | 518 |
| **51** | Beatty$.tw. | 58 |
| **52** | Braly$.tw. | 10 |
| **53** | Hazel$.tw. | 914 |
| **54** | mfer.tw. | 7 |
| **55** | Maximum flexion external rotation.tw. | 1 |
| **56** | mfir.tw. | 11 |
| **57** | maximum flexion internal rotation.tw. | 2 |
| **58** | Morris$.tw. | 6877 |
| **59** | Nachlas$.tw. | 29 |
| **60** | nelaton$.tw. | 70 |
| **61** | neri$.tw. | 624 |
| **62** | bowing.tw. | 967 |
| **63** | noble$.tw. | 2114 |
| **64** | noble compression.tw. | 0 |
| **65** | ober$.tw. | 783 |
| **66** | ortolani$.tw. | 117 |
| **67** | Pace$.tw. | 39204 |
| **68** | pace sign.tw. | 0 |
| **69** | Phelps$.tw. | 104 |
| **70** | psoas strength.tw. | 0 |
| **71** | quadriceps flexion.tw. | 0 |
| **72** | single straight leg raise.tw. | 0 |
| **73** | shoemaker$.tw. | 146 |
| **74** | Stinchfield$.tw. | 31 |
| **75** | thomas$.tw. | 6236 |
| **76** | thomas test.tw. | 25 |
| **77** | torque test.tw. | 50 |
| **78** | trendelenburg$.tw. | 947 |
| **79** | tripod$.tw. | 745 |
| **80** | Seated Straight Leg Raise.tw. | 1 |
| **81** | or/1-80 | 169415 |
| **82** | exp physical examination/ | 847042 |
| **83** | exp Hip Joint/ or exp Hip/ or exp hip injuries/ | 35849 |
| **84** | 82 and 83 | 4147 |
| **85** | ((clinical or physical) adj2 (exam$ or sign$ or test$ or evaluation or investigation or diagnosis or analysis)).tw. | 247026 |
| **86** | (objective adj2 exam$).tw. | 20057 |
| **87** | (special adj2 test$).tw. | 985 |
| **88** | 85 or 86 or 87 | 266982 |
| **89** | (hip or hips or hip joint or pelvis or groin or femur or femoral or acetabul$ or trochanter or iliofemoral or ischiofemoral or pubofemoral or iliopsoas or sartorius or quadriceps or rectus femoris or tensor fascia lat?e or pectineus or adductor brevis or adductor longus or adductor magnus or gluteus medius or gluteus minimus or gluteus maximus or glute$ or hamstrings or adductors or adductor magnus or adductor longus or adductor brevis or gracilis or obturator internus or obturator externus or gemell$ or quadratus femoris or piriformis or semitendinosus or semimembranosus or biceps femoris or iliacus or obturator or iliotibial).tw. | 199253 |
| **90** | 88 and 89 | 6947 |
| **91** | 84 or 90 | 10860 |
| **92** | limit 91 to (english language and humans) | 8498 |
| **93** | 81 and 84 | 174 |
| **94** | 81 and 88 | 3198 |
| **95** | 81 and 89 | 2863 |
| **96** | or/93-95 | 5865 |
| **97** | limit 96 to (english language and humans) | 4424 |
| **98** | 92 or 97 | 12628 |

Embase (1988 to July 2010) via Ovid. This search was conducted on 20/07/2010.

| **No.** | **Search Terms** | **Results** |
| --- | --- | --- |
| **1** | actual leg length.tw. | 3 |
| **2** | allis$.tw. | 159 |
| **3** | galeazzi$.tw. | 99 |
| **4** | anvil$.tw. | 268 |
| **5** | heel strike.tw. | 377 |
| **6** | apparent leg length.tw. | 7 |
| **7** | Barlow$.tw. | 228 |
| **8** | berry$.tw. | 834 |
| **9** | Bryant$.tw. | 228 |
| **10** | buttock sign.tw. | 1 |
| **11** | Chiene$.tw. | 2 |
| **12** | Craig$.tw. | 442 |
| **13** | Dial$.tw. | 67383 |
| **14** | ely$.tw. | 399 |
| **15** | heel to buttock.tw. | 2 |
| **16** | faber$.tw. | 141 |
| **17** | Patrick$.tw. | 229 |
| **18** | Flexion, Abduction External Rotation.tw. | 15 |
| **19** | Figure four.tw. | 4 |
| **20** | faddir.tw. | 0 |
| **21** | flexion adduction.mp. and internal rotation.tw. [mp=title, abstract, subject headings, heading word, drug trade name, original title, device manufacturer, drug manufacturer name] | 23 |
| **22** | Fitzgerald$.tw. | 141 |
| **23** | Flexion Abduction.mp. and Internal Rotation.tw. [mp=title, abstract, subject headings, heading word, drug trade name, original title, device manufacturer, drug manufacturer name] | 35 |
| **24** | Flexion Adduction Axial Compression.tw. | 0 |
| **25** | Flexion Internal Rotation Axial Compression.tw. | 0 |
| **26** | Freiberg$.tw. | 89 |
| **27** | Fulcrum$.tw. | 269 |
| **28** | Gauvain$.tw. | 2 |
| **29** | Hamstring 90.tw. | 0 |
| **30** | straight leg raising.tw. | 290 |
| **31** | Hanneqin$.tw. | 0 |
| **32** | Hibb$.tw. | 55 |
| **33** | hip abduction stress.tw. | 0 |
| **34** | hip adduction deformity.tw. | 1 |
| **35** | Scour$.tw. | 658 |
| **36** | telescop$.tw. | 1471 |
| **37** | hop test.tw. | 102 |
| **38** | impingement test.tw. | 44 |
| **39** | Flexion Adduction Internal Rotation.tw. | 3 |
| **40** | impingement provocation.tw. | 0 |
| **41** | Jansen$.tw. | 324 |
| **42** | Laguerre$.tw. | 61 |
| **43** | Lasegue$.tw. | 95 |
| **44** | Lewin$.tw. | 168 |
| **45** | log roll.tw. | 18 |
| **46** | log roll test.tw. | 1 |
| **47** | Ludloff$.tw. | 41 |
| **48** | Lunge$.tw. | 187 |
| **49** | Malinger$.tw. | 1039 |
| **50** | McCarthy$.tw. | 308 |
| **51** | Beatty$.tw. | 25 |
| **52** | Braly$.tw. | 0 |
| **53** | Hazel$.tw. | 698 |
| **54** | mfer.tw. | 5 |
| **55** | Maximum flexion external rotation.tw. | 1 |
| **56** | mfir.tw. | 8 |
| **57** | maximum flexion internal rotation.tw. | 2 |
| **58** | Morris$.tw. | 5475 |
| **59** | Nachlas$.tw. | 6 |
| **60** | nelaton$.tw. | 43 |
| **61** | neri$.tw. | 559 |
| **62** | bowing.tw. | 659 |
| **63** | noble$.tw. | 1344 |
| **64** | noble compression.tw. | 0 |
| **65** | ober$.tw. | 696 |
| **66** | ortolani$.tw. | 62 |
| **67** | Pace$.tw. | 25945 |
| **68** | pace sign.tw. | 0 |
| **69** | Phelps$.tw. | 58 |
| **70** | psoas strength.tw. | 0 |
| **71** | quadriceps flexion.tw. | 0 |
| **72** | single straight leg raise.tw. | 0 |
| **73** | shoemaker$.tw. | 109 |
| **74** | Stinchfield$.tw. | 10 |
| **75** | thomas$.tw. | 3697 |
| **76** | thomas test.tw. | 25 |
| **77** | torque test.tw. | 26 |
| **78** | trendelenburg$.tw. | 654 |
| **79** | tripod$.tw. | 700 |
| **80** | Seated Straight Leg Raise.tw. | 1 |
| **81** | or/1-80 | 116562 |
| **82** | exp physical examination/ | 81698 |
| **83** | exp Hip Joint/ or exp Hip/ or exp hip injuries/ | 23677 |
| **84** | 82 and 83 | 467 |
| **85** | ((clinical or physical) adj2 (exam$ or sign$ or test$ or evaluation or investigation or diagnosis or analysis)).tw. | 176169 |
| **86** | (objective adj2 exam$).tw. | 17707 |
| **87** | (special adj2 test$).tw. | 540 |
| **88** | 85 or 86 or 87 | 193476 |
| **89** | (hip or hips or hip joint or pelvis or groin or femur or femoral or acetabul$ or trochanter or iliofemoral or ischiofemoral or pubofemoral or iliopsoas or sartorius or quadriceps or rectus femoris or tensor fascia lat?e or pectineus or adductor brevis or adductor longus or adductor magnus or gluteus medius or gluteus minimus or gluteus maximus or glute$ or hamstrings or adductors or adductor magnus or adductor longus or adductor brevis or gracilis or obturator internus or obturator externus or gemell$ or quadratus femoris or piriformis or semitendinosus or semimembranosus or biceps femoris or iliacus or obturator or iliotibial).tw. | 138687 |
| **90** | 88 and 89 | 5777 |
| **91** | 84 or 90 | 6101 |
| **92** | limit 91 to (english language and humans) | 4723 |
| **93** | 81 and 84 | 17 |
| **94** | 81 and 88 | 2561 |
| **95** | 81 and 89 | 2194 |
| **96** | or/93-95 | 4582 |
| **97** | limit 96 to (english language and humans) | 3530 |
| **98** | 92 or 97 | 8099 |

Embase Classic (1947-1979) via Ovid. This search was conducted on 20/07/2010.

| **No.** | **Search Terms** | **Results** |
| --- | --- | --- |
| **1** | actual leg length.tw. | 1 |
| **2** | allis$.tw. | 183 |
| **3** | galeazzi$.tw. | 37 |
| **4** | anvil$.tw. | 39 |
| **5** | heel strike.tw. | 34 |
| **6** | apparent leg length.tw. | 0 |
| **7** | Barlow$.tw. | 92 |
| **8** | berry$.tw. | 249 |
| **9** | Bryant$.tw. | 44 |
| **10** | buttock sign.tw. | 0 |
| **11** | Chiene$.tw. | 0 |
| **12** | Craig$.tw. | 178 |
| **13** | Dial$.tw. | 17358 |
| **14** | ely$.tw. | 106 |
| **15** | heel to buttock.tw. | 0 |
| **16** | faber$.tw. | 75 |
| **17** | Patrick$.tw. | 21 |
| **18** | Flexion, Abduction External Rotation.tw. | 1 |
| **19** | Figure four.tw. | 4 |
| **20** | faddir.tw. | 0 |
| **21** | flexion adduction.mp. and internal rotation.tw. [mp=title, abstract, subject headings, heading word, drug trade name, original title, device manufacturer, drug manufacturer name] | 3 |
| **22** | Fitzgerald$.tw. | 128 |
| **23** | Flexion Abduction.mp. and Internal Rotation.tw. [mp=title, abstract, subject headings, heading word, drug trade name, original title, device manufacturer, drug manufacturer name] | 3 |
| **24** | Flexion Adduction Axial Compression.tw. | 0 |
| **25** | Flexion Internal Rotation Axial Compression.tw. | 0 |
| **26** | Freiberg$.tw. | 23 |
| **27** | Fulcrum$.tw. | 97 |
| **28** | Gauvain$.tw. | 0 |
| **29** | Hamstring 90.tw. | 0 |
| **30** | straight leg raising.tw. | 71 |
| **31** | Hanneqin$.tw. | 0 |
| **32** | Hibb$.tw. | 70 |
| **33** | hip abduction stress.tw. | 0 |
| **34** | hip adduction deformity.tw. | 0 |
| **35** | Scour$.tw. | 300 |
| **36** | telescop$.tw. | 508 |
| **37** | hop test.tw. | 0 |
| **38** | impingement test.tw. | 0 |
| **39** | Flexion Adduction Internal Rotation.tw. | 0 |
| **40** | impingement provocation.tw. | 0 |
| **41** | Jansen$.tw. | 141 |
| **42** | Laguerre$.tw. | 10 |
| **43** | Lasegue$.tw. | 87 |
| **44** | Lewin$.tw. | 135 |
| **45** | log roll.tw. | 0 |
| **46** | log roll test.tw. | 0 |
| **47** | Ludloff$.tw. | 19 |
| **48** | Lunge$.tw. | 235 |
| **49** | Malinger$.tw. | 384 |
| **50** | McCarthy$.tw. | 90 |
| **51** | Beatty$.tw. | 31 |
| **52** | Braly$.tw. | 5 |
| **53** | Hazel$.tw. | 228 |
| **54** | mfer.tw. | 0 |
| **55** | Maximum flexion external rotation.tw. | 0 |
| **56** | mfir.tw. | 1 |
| **57** | maximum flexion internal rotation.tw. | 0 |
| **58** | Morris$.tw. | 884 |
| **59** | Nachlas$.tw. | 93 |
| **60** | nelaton$.tw. | 74 |
| **61** | neri$.tw. | 201 |
| **62** | bowing.tw. | 290 |
| **63** | noble$.tw. | 474 |
| **64** | noble compression.tw. | 0 |
| **65** | ober$.tw. | 456 |
| **66** | ortolani$.tw. | 62 |
| **67** | Pace$.tw. | 7246 |
| **68** | pace sign.tw. | 0 |
| **69** | Phelps$.tw. | 56 |
| **70** | psoas strength.tw. | 0 |
| **71** | quadriceps flexion.tw. | 0 |
| **72** | single straight leg raise.tw. | 0 |
| **73** | shoemaker$.tw. | 61 |
| **74** | Stinchfield$.tw. | 1 |
| **75** | thomas$.tw. | 984 |
| **76** | thomas test.tw. | 3 |
| **77** | torque test.tw. | 0 |
| **78** | trendelenburg$.tw. | 625 |
| **79** | tripod$.tw. | 91 |
| **80** | Seated Straight Leg Raise.tw. | 0 |
| **81** | or/1-80 | 32462 |
| **82** | exp physical examination/ | 5852 |
| **83** | exp Hip Joint/ or exp Hip/ or exp hip injuries/ | 12541 |
| **84** | 82 and 83 | 59 |
| **85** | ((clinical or physical) adj2 (exam$ or sign$ or test$ or evaluation or investigation or diagnosis or analysis)).tw. | 47389 |
| **86** | (objective adj2 exam$).tw. | 277 |
| **87** | (special adj2 test$).tw. | 553 |
| **88** | or/85-87 | 48138 |
| **89** | (hip or hips or hip joint or pelvis or groin or femur or femoral or acetabul$ or trochanter or iliofemoral or ischiofemoral or pubofemoral or iliopsoas or sartorius or quadriceps or rectus femoris or tensor fascia lat?e or pectineus or adductor brevis or adductor longus or adductor magnus or gluteus medius or gluteus minimus or gluteus maximus or glute$ or hamstrings or adductors or adductor magnus or adductor longus or adductor brevis or gracilis or obturator internus or obturator externus or gemell$ or quadratus femoris or piriformis or semitendinosus or semimembranosus or biceps femoris or iliacus or obturator or iliotibial).tw. | 49028 |
| **90** | 88 and 89 | 1062 |
| **91** | 84 or 90 | 1092 |
| **92** | limit 91 to (english language and humans) | 30 |
| **93** | 81 and 84 | 2 |
| **94** | 81 and 88 | 500 |
| **95** | 81 and 89 | 728 |
| **96** | or/93-95 | 1205 |
| **97** | limit 96 to (english language and humans) | 57 |
| **98** | 92 or 97 | 87 |

CINAHL via EBSCO (1983-2010). This search was conducted on 20/07/2010.

| **No.** | **Search Terms** | **Results** |
| --- | --- | --- |
| **S1** | (MH "Physical Examination+") | 40932 |
| **S2** | (MH "Hip") or (MH "Hip Injuries+") or (MH "Hip Joint") | 6973 |
| **S3** | S1 and S2 | 500 |
| **S4** | (clinical n2 exam*) or (clinical n2 sign*) or (clinical n2 test*) or (clinical n2 evaluation) or (clinical n2 investigation) or (clinical n2 diagnosis) or (clinical n2 analysis) or (physical n2 exam*) or (physical n2 sign*) or (physical n2 test*) or (physical n2 evaluation) or (physical n2 investigation) or (physical n2 diagnosis) or (physical n2 analysis) | 45066 |
| **S5** | objective n2 exam* | 7069 |
| **S6** | special n2 test* | 146 |
| **S7** | S4 or S5 or S6 | 51504 |
| **S8** | hip or hips or hip joint or pelvis or groin or femur or femoral or acetabul* or trochanter or iliofemoral or ischiofemoral or pubofemoral or iliopsoas or sartorius or quadriceps or rectus femoris or tensor fascia lat?e or pectineus or adductor brevis or adductor longus or adductor magnus or gluteus medius or gluteus minimus or gluteus maximus or glute* or hamstrings or adductors or adductor magnus or adductor longus or adductor brevis or gracilis or obturator internus or obturator externus or gemell* or quadratus femoris or piriformis or semitendinosus or semimembranosus or biceps femoris or iliacus or obturator or iliotibial | 27025 |
| **S9** | S7 and S8 | 2070 |
| **S10** | S3 or S9 | 2323 |
| **S11** | **Limiters** - English Language; Human | 1562 |

**Part II: Additional Physical Tests**

Medline (1950 to November 2010) via Ovid. This search was conducted on 21/11/2010.

| **No.** | **Search Terms** | **Results** |
| --- | --- | --- |
| **1** | percuss$.tw. and exp hip fracture/ | 4 |
| **2** | (apprehension test and capsul$).tw. | 15 |
| **3** | (axial distraction and capsul$).tw. | 0 |
| **4** | (prone hip extension and contracture$).tw. | 2 |
| **5** | (staheli and contracture$).tw. | 0 |
| **6** | (iliacus test and hip$).tw. | 1 |
| **7** | (taking off the shoe and biceps femoris).tw. | 1 |
| **8** | (resisted range of motion and biceps femoris).tw. | 1 |
| **9** | (antalgic gait and hip$).tw. | 14 |
| **10** | (femoral stretch test and hip$).tw. | 0 |
| **11** | passive rotation.tw. and exp hip fractures/ | 0 |
| **12** | (single leg stance and $bursitis).tw. | 1 |
| **13** | (resisted external derotation and hip$).tw. | 1 |
| **14** | (internal rotation over pressure and hip$).tw. | 1 |
| **15** | manual muscle test.tw. and exp Osteoarthritis/ | 2 |
| **16** | (Duncan test and rectus femoris).tw. | 0 |
| **17** | (duncan ely and rectus femoris).tw. | 7 |
| **18** | (external rotation abduction and $bursitis).tw. | 0 |
| **19** | or/1-18 | 48 |
| **20** | limit 19 to english language | 44 |

Embase (1988 to November 2010) via Ovid. This search was conducted on 21/11/2010.

| **No.** | **Search Terms** | **Results** |
| --- | --- | --- |
| **1** | percuss$.tw. and exp hip fracture/ | 3 |
| **2** | (apprehension test and capsul$).tw. | 15 |
| **3** | (axial distraction and capsul$).tw. | 1 |
| **4** | (prone hip extension and contracture$).tw. | 0 |
| **5** | (staheli and contracture$).tw. | 0 |
| **6** | (iliacus test and hip$).tw. | 1 |
| **7** | (taking off the shoe and biceps femoris).tw. | 1 |
| **8** | (resisted range of motion and biceps femoris).tw. | 1 |
| **9** | (antalgic gait and hip$).tw. | 16 |
| **10** | (femoral stretch test and hip$).tw. | 0 |
| **11** | passive rotation.tw. and exp hip fractures/ | 0 |
| **12** | (single leg stance and $bursitis).tw. | 1 |
| **13** | (resisted external derotation and hip$).tw. | 1 |
| **14** | (internal rotation over pressure and hip$).tw. | 1 |
| **15** | manual muscle test.tw. and exp Osteoarthritis/ | 2 |
| **16** | (Duncan test and rectus femoris).tw. | 0 |
| **17** | (duncan ely and rectus femoris).tw. | 10 |
| **18** | (external rotation abduction and $bursitis).tw. | 0 |
| **19** | or/1-18 | 50 |
| **20** | limit 19 to english language | 45 |

Embase Classic (1947-1979) via Ovid. This search was conducted on 21/11/2010.

| **No.** | **Search Terms** | **Results** |
| --- | --- | --- |
| **1** | percuss$.tw. and exp hip fracture/ | 0 |
| **2** | (apprehension test and capsul$).tw. | 0 |
| **3** | (axial distraction and capsul$).tw. | 0 |
| **4** | (prone hip extension and contracture$).tw. | 0 |
| **5** | (staheli and contracture$).tw. | 0 |
| **6** | (iliacus test and hip$).tw. | 0 |
| **7** | (taking off the shoe and biceps femoris).tw. | 0 |
| **8** | (resisted range of motion and biceps femoris).tw. | 0 |
| **9** | (antalgic gait and hip$).tw. | 2 |
| **10** | (femoral stretch test and hip$).tw. | 0 |
| **11** | passive rotation.tw. and exp hip fractures/ | 0 |
| **12** | (single leg stance and $bursitis).tw. | 0 |
| **13** | (resisted external derotation and hip$).tw. | 0 |
| **14** | (internal rotation over pressure and hip$).tw. | 0 |
| **15** | manual muscle test.tw. and exp Osteoarthritis/ | 0 |
| **16** | (Duncan test and rectus femoris).tw. | 0 |
| **17** | (duncan ely and rectus femoris).tw. | 0 |
| **18** | (external rotation abduction and $bursitis).tw. | 0 |
| **19** | or/1-18 | 2 |
| **20** | limit 19 to english language | 2 |

Medline (1950 to November 2010) via Ovid. This search was conducted on 23/11/2010.

| **No.** | **Search Terms** | **Results** |
| --- | --- | --- |
| **1** | Cyriax.tw. | 27 |
| **2** | exp Osteoarthritis, Hip/ or exp Osteoarthritis/ | 35597 |
| **3** | 1 and 2 | 4 |
| **4** | Kaltenborn.tw. | 5 |
| **5** | 2 and 4 | 1 |
| **6** | 3 or 5 | 5 |
| **7** | limit 6 to (english language and humans) | 5 |

Embase (1988 to November 2010) via Ovid. This search was conducted on 23/11/2010.

| **No.** | **Search Terms** | **Results** |
| --- | --- | --- |
| **1** | Cyriax.tw. | 40 |
| **2** | exp Osteoarthritis, Hip/ or exp Osteoarthritis/ | 45824 |
| **3** | 1 and 2 | 3 |
| **4** | Kaltenborn.tw. | 7 |
| **5** | 2 and 4 | 1 |
| **6** | 3 or 5 | 4 |
| **7** | limit 6 to (english language and humans) | 4 |

Embase Classic (1947-1979) via Ovid. This search was conducted on 23/11/2010.

| **No.** | **Search Terms** | **Results** |
| --- | --- | --- |
| **1** | Cyriax.tw. | 13 |
| **2** | exp Osteoarthritis, Hip/ or exp Osteoarthritis/ | 9488 |
| **3** | 1 and 2 | 0 |
| **4** | Kaltenborn.tw. | 2 |
| **5** | 2 and 4 | 1 |
| **6** | 3 or 5 | 1 |
| **7** | limit 6 to (english language and humans) | 0 |

Medline (1950 to January 2011) via Ovid. This search was conducted on 1/1/2011.

| **No.** | **Search Terms** | **Results** |
| --- | --- | --- |
| **1** | Active squat test.tw. | 0 |
| **2** | hip flexion test.tw. | 4 |
| **3** | Single Leg Stance Phase.tw. | 5 |
| **4** | Dynamic External Rotatory Impingement.tw. | 0 |
| **5** | Dynamic Internal Rotatory Impingement.tw. | 0 |
| **6** | Tensor Fascia$ Lata$ Contracture.tw. | 0 |
| **7** | Gluteus Medius Contracture Test.tw. | 0 |
| **8** | Femoral Anteversion Test.tw. | 1 |
| **9** | Quadrant Test.tw. | 6 |
| **10** | Hyperlordosis Sign.tw. | 1 |
| **11** | Developpe.tw. | 1 |
| **12** | Hip Abduction sign.tw. | 1 |
| **13** | or/1-12 | 18 |
| **14** | limit 13 to (english language and humans) | 17 |

Embase (1988 to January 2011) via Ovid. This search was conducted on 1/1/2011.

| **No.** | **Search Terms** | **Results** |
| --- | --- | --- |
| **1** | Active squat test.tw. | 0 |
| **2** | hip flexion test.tw. | 4 |
| **3** | Single Leg Stance Phase.tw. | 5 |
| **4** | Dynamic External Rotatory Impingement.tw. | 0 |
| **5** | Dynamic Internal Rotatory Impingement.tw. | 0 |
| **6** | Tensor Fascia$ Lata$ Contracture.tw. | 0 |
| **7** | Gluteus Medius Contracture Test.tw. | 0 |
| **8** | Femoral Anteversion Test.tw. | 1 |
| **9** | Quadrant Test.tw. | 9 |
| **10** | Hyperlordosis Sign.tw. | 0 |
| **11** | Developpe.tw. | 10 |
| **12** | Hip Abduction sign.tw. | 2 |
| **13** | or/1-12 | 30 |
| **14** | limit 13 to (english language and humans) | 20 |

Embase Classic (1947-1979) via Ovid. This search was conducted on 1/1/2011.

| **No.** | **Search Terms** | **Results** |
| --- | --- | --- |
| **1** | Active squat test.tw. | 0 |
| **2** | hip flexion test.tw. | 0 |
| **3** | Single Leg Stance Phase.tw. | 0 |
| **4** | Dynamic External Rotatory Impingement.tw. | 0 |
| **5** | Dynamic Internal Rotatory Impingement.tw. | 0 |
| **6** | Tensor Fascia$ Lata$ Contracture.tw. | 1 |
| **7** | Gluteus Medius Contracture Test.tw. | 0 |
| **8** | Femoral Anteversion Test.tw. | 0 |
| **9** | Quadrant Test.tw. | 0 |
| **10** | Hyperlordosis Sign.tw. | 0 |
| **11** | Developpe.tw. | 0 |
| **12** | Hip Abduction sign.tw. | 0 |
| **13** | or/1-12 | 1 |
| **14** | limit 13 to (english language and humans) | 0 |

**Part III: Repeated Search Strategy in 2013**.

Medline to March 2013 via Ovid. This search was conducted on 03/03/2013.

| **No.** | **Search Terms** | **Results** |
| --- | --- | --- |
| **1** | actual leg length.tw. | 3 |
| **2** | allis$.tw. | 171 |
| **3** | galeazzi$.tw. | 83 |
| **4** | anvil$.tw. | 297 |
| **5** | heel strike.tw. | 388 |
| **6** | apparent leg length.tw. | 5 |
| **7** | Barlow$.tw. | 238 |
| **8** | berry$.tw. | 1469 |
| **9** | Bryant$.tw. | 220 |
| **10** | buttock sign.tw. | 0 |
| **11** | Chiene$.tw. | 0 |
| **12** | Craig$.tw. | 531 |
| **13** | Dial$.tw. | 63792 |
| **14** | ely$.tw. | 507 |
| **15** | heel to buttock.tw. | 1 |
| **16** | faber$.tw. | 128 |
| **17** | Patrick$.tw. | 353 |
| **18** | Flexion, Abduction External Rotation.tw. | 25 |
| **19** | Figure four.tw. | 3 |
| **20** | faddir.tw. | 0 |
| **21** | flexion adduction.mp. and internal rotation.tw. | 30 |
| **22** | Fitzgerald$.tw. | 130 |
| **23** | Flexion Abduction.mp. and Internal Rotation.tw. | 49 |
| **24** | Flexion Adduction Axial Compression.tw. | 0 |
| **25** | Flexion Internal Rotation Axial Compression.tw. | 0 |
| **26** | Freiberg$.tw. | 88 |
| **27** | Fulcrum$.tw. | 301 |
| **28** | Gauvain$.tw. | 0 |
| **29** | Hamstring 90.tw. | 0 |
| **30** | straight leg raising.tw. | 204 |
| **31** | Hanneqin$.tw. | 0 |
| **32** | Hibb$.tw. | 38 |
| **33** | hip abduction stress.tw. | 0 |
| **34** | hip adduction deformity.tw. | 0 |
| **35** | Scour$.tw. | 776 |
| **36** | telescop$.tw. | 1474 |
| **37** | hop test.tw. | 152 |
| **38** | impingement test.tw. | 58 |
| **39** | Flexion Adduction Internal Rotation.tw. | 6 |
| **40** | impingement provocation.tw. | 1 |
| **41** | Jansen$.tw. | 146 |
| **42** | Laguerre$.tw. | 129 |
| **43** | Lasegue$.tw. | 71 |
| **44** | Lewin$.tw. | 220 |
| **45** | log roll.tw. | 30 |
| **46** | log roll test.tw. | 2 |
| **47** | Ludloff$.tw. | 34 |
| **48** | Lunge$.tw. | 236 |
| **49** | Malinger$.tw. | 858 |
| **50** | McCarthy$.tw. | 273 |
| **51** | Beatty$.tw. | 23 |
| **52** | Braly$.tw. | 5 |
| **53** | Hazel$.tw. | 790 |
| **54** | mfer.tw. | 7 |
| **55** | Maximum flexion external rotation.tw. | 1 |
| **56** | mfir.tw. | 9 |
| **57** | maximum flexion internal rotation.tw. | 2 |
| **58** | Morris$.tw. | 6055 |
| **59** | Nachlas$.tw. | 3 |
| **60** | nelaton$.tw. | 46 |
| **61** | neri$.tw. | 527 |
| **62** | bowing.tw. | 634 |
| **63** | noble$.tw. | 1800 |
| **64** | noble compression.tw. | 0 |
| **65** | ober$.tw. | 569 |
| **66** | ober$.tw. | 569 |
| **67** | Pace$.tw. | 25583 |
| **68** | pace sign.tw. | 0 |
| **69** | Phelps$.tw. | 59 |
| **70** | psoas strength.tw. | 0 |
| **71** | quadriceps flexion.tw. | 0 |
| **72** | single straight leg raise.tw. | 0 |
| **73** | shoemaker$.tw. | 78 |
| **74** | Stinchfield$.tw. | 23 |
| **75** | thomas$.tw. | 3457 |
| **76** | thomas test.tw. | 27 |
| **77** | torque test.tw. | 46 |
| **78** | trendelenburg$.tw. | 652 |
| **79** | tripod$.tw. | 721 |
| **80** | Seated Straight Leg Raise.tw. | 1 |
| **81** | percuss$.tw. | 1615 |
| **82** | apprehension test.tw. | 67 |
| **83** | axial distraction.tw. | 27 |
| **84** | prone hip extension.tw. | 12 |
| **85** | staheli.tw. | 18 |
| **86** | iliacus test.tw. | 1 |
| **87** | taking off the shoe.tw. | 1 |
| **88** | resisted range of motion.tw. | 1 |
| **89** | antalgic gait.tw. | 27 |
| **90** | femoral stretch test.tw. | 5 |
| **91** | passive rotation.tw. | 62 |
| **92** | single leg stance.tw. | 223 |
| **93** | resisted external derotation.tw. | 1 |
| **94** | internal rotation over pressure.tw. | 1 |
| **95** | manual muscle test.tw. | 113 |
| **96** | Duncan test.tw. | 76 |
| **97** | duncan ely.tw. | 8 |
| **98** | external rotation abduction.tw. | 24 |
| **99** | or/1-98 | 116249 |
| **100** | exp physical examination/ | 479338 |
| **101** | exp Hip Joint/ or exp Hip/ or exp hip injuries/ | 19481 |
| **102** | 100 and 101 | 3667 |
| **103** | ((clinical or physical) adj2 (exam$ or sign$ or test$ or evaluation or investigation or diagnosis or analysis)).tw. | 183156 |
| **104** | (objective adj2 exam$).tw. | 21939 |
| **105** | (special adj2 test$).tw. | 470 |
| **106** | 103 or 104 or 105 | 204524 |
| **107** | (hip or hips or hip joint or pelvis or groin or femur or femoral or acetabul$ or trochanter or iliofemoral or ischiofemoral or pubofemoral or iliopsoas or sartorius or quadriceps or rectus femoris or tensor fascia lat?e or pectineus or adductor brevis or adductor longus or adductor magnus or gluteus medius or gluteus minimus or gluteus maximus or glute$ or hamstrings or adductors or adductor magnus or adductor longus or adductor brevis or gracilis or obturator internus or obturator externus or gemell$ or quadratus femoris or piriformis or semitendinosus or semimembranosus or biceps femoris or iliacus or obturator or iliotibial).tw. | 136433 |
| **108** | 106 and 107 | 6076 |
| **109** | 102 or 108 | 9494 |
| **110** | limit 109 to (english language and humans) | 7867 |
| **111** | 99 and 102 | 192 |
| **112** | 99 and 106 | 2889 |
| **113** | 99 and 107 | 2447 |
| **114** | or/111-113 | 5139 |
| **115** | limit 114 to (english language and humans) | 4209 |
| **116** | 110 or 115 | 11746 |
| **117** | limit 116 to yr=”2010 –Current” | 3054 |

Embase to March 2013 via Ovid. This search was conducted on 03/03/2013.

| **No.** | **Search Terms** | **Results** |
| --- | --- | --- |
| **1** | actual leg length.tw. | 4 |
| **2** | allis$.tw. | 272 |
| **3** | galeazzi$.tw. | 117 |
| **4** | anvil$.tw. | 522 |
| **5** | heel strike.tw. | 500 |
| **6** | apparent leg length.tw. | 10 |
| **7** | Barlow$.tw. | 358 |
| **8** | berry$.tw. | 2085 |
| **9** | Bryant$.tw. | 301 |
| **10** | buttock sign.tw. | 0 |
| **11** | Chiene$.tw. | 2 |
| **12** | Craig$.tw. | 792 |
| **13** | Dial$.tw. | 92423 |
| **14** | ely$.tw. | 687 |
| **15** | heel to buttock.tw. | 4 |
| **16** | faber$.tw. | 201 |
| **17** | Patrick$.tw. | 510 |
| **18** | Flexion, Abduction External Rotation.tw. | 32 |
| **19** | Figure four.tw. | 11 |
| **20** | faddir.tw. | 0 |
| **21** | flexion adduction.mp. and internal rotation.tw. | 39 |
| **22** | Fitzgerald$.tw. | 227 |
| **23** | Flexion Abduction.mp. and Internal Rotation.tw. | 67 |
| **24** | Flexion Adduction Axial Compression.tw. | 0 |
| **25** | Flexion Internal Rotation Axial Compression.tw. | 0 |
| **26** | Freiberg$.tw. | 134 |
| **27** | Fulcrum$.tw. | 426 |
| **28** | Gauvain$.tw. | 2 |
| **29** | Hamstring 90.tw. | 0 |
| **30** | straight leg raising.tw. | 287 |
| **31** | Hanneqin$.tw. | 0 |
| **32** | Hibb$.tw. | 62 |
| **33** | hip abduction stress.tw. | 0 |
| **34** | hip adduction deformity.tw. | 0 |
| **35** | Scour$.tw. | 1081 |
| **36** | telescop$.tw. | 2323 |
| **37** | hop test.tw. | 182 |
| **38** | impingement test.tw. | 75 |
| **39** | Flexion Adduction Internal Rotation.tw. | 8 |
| **40** | impingement provocation.tw. | 1 |
| **41** | Jansen$.tw. | 427 |
| **42** | Laguerre$.tw. | 152 |
| **43** | Lasegue$.tw. | 113 |
| **44** | Lewin$.tw. | 297 |
| **45** | log roll.tw. | 38 |
| **46** | log roll test.tw. | 3 |
| **47** | Ludloff$.tw. | 48 |
| **48** | Lunge$.tw. | 379 |
| **49** | Malinger$.tw. | 1286 |
| **50** | McCarthy$.tw. | 422 |
| **51** | Beatty$.tw. | 38 |
| **52** | Braly$.tw. | 4 |
| **53** | Hazel$.tw. | 1229 |
| **54** | mfer.tw. | 10 |
| **55** | Maximum flexion external rotation.tw. | 1 |
| **56** | mfir.tw. | 24 |
| **57** | maximum flexion internal rotation.tw. | 2 |
| **58** | Morris$.tw. | 8746 |
| **59** | Nachlas$.tw. | 3 |
| **60** | nelaton$.tw. | 81 |
| **61** | neri$.tw. | 880 |
| **62** | bowing.tw. | 882 |
| **63** | noble$.tw. | 2656 |
| **64** | noble compression.tw. | 0 |
| **65** | ober$.tw. | 1053 |
| **66** | ober$.tw. | 1053 |
| **67** | Pace$.tw. | 37537 |
| **68** | pace sign.tw. | 1 |
| **69** | Phelps$.tw. | 79 |
| **70** | psoas strength.tw. | 0 |
| **71** | quadriceps flexion.tw. | 0 |
| **72** | single straight leg raise.tw. | 0 |
| **73** | shoemaker$.tw. | 133 |
| **74** | Stinchfield$.tw. | 29 |
| **75** | thomas$.tw. | 6010 |
| **76** | thomas test.tw. | 36 |
| **77** | torque test.tw. | 45 |
| **78** | trendelenburg$.tw. | 1044 |
| **79** | tripod$.tw. | 1292 |
| **80** | Seated Straight Leg Raise.tw. | 1 |
| **81** | percuss$.tw. | 2351 |
| **82** | apprehension test.tw. | 82 |
| **83** | axial distraction.tw. | 30 |
| **84** | prone hip extension.tw. | 15 |
| **85** | staheli.tw. | 28 |
| **86** | iliacus test.tw. | 1 |
| **87** | taking off the shoe.tw. | 1 |
| **88** | resisted range of motion.tw. | 1 |
| **89** | antalgic gait.tw. | 47 |
| **90** | femoral stretch test.tw. | 7 |
| **91** | passive rotation.tw. | 77 |
| **92** | single leg stance.tw. | 293 |
| **93** | resisted external derotation.tw. | 1 |
| **94** | internal rotation over pressure.tw. | 1 |
| **95** | manual muscle test.tw. | 163 |
| **96** | Duncan test.tw. | 122 |
| **97** | duncan ely.tw. | 12 |
| **98** | external rotation abduction.tw. | 29 |
| **99** | or/1-98 | 170957 |
| **100** | exp physical examination/ | 118843 |
| **101** | exp Hip Joint/ or exp Hip/ or exp hip injuries/ | 38714 |
| **102** | 100 and 101 | 869 |
| **103** | ((clinical or physical) adj2 (exam$ or sign$ or test$ or evaluation or investigation or diagnosis or analysis)).tw. | 277524 |
| **104** | (objective adj2 exam$).tw. | 26294 |
| **105** | (special adj2 test$).tw. | 714 |
| **106** | 103 or 104 or 105 | 303125 |
| **107** | (hip or hips or hip joint or pelvis or groin or femur or femoral or acetabul$ or trochanter or iliofemoral or ischiofemoral or pubofemoral or iliopsoas or sartorius or quadriceps or rectus femoris or tensor fascia lat?e or pectineus or adductor brevis or adductor longus or adductor magnus or gluteus medius or gluteus minimus or gluteus maximus or glute$ or hamstrings or adductors or adductor magnus or adductor longus or adductor brevis or gracilis or obturator internus or obturator externus or gemell$ or quadratus femoris or piriformis or semitendinosus or semimembranosus or biceps femoris or iliacus or obturator or iliotibial).tw. | 197511 |
| **108** | 106 and 107 | 9703 |
| **109** | 102 or 108 | 10219 |
| **110** | limit 109 to (english language and humans) | 7313 |
| **111** | 99 and 102 | 51 |
| **112** | 99 and 106 | 4637 |
| **113** | 99 and 107 | 3856 |
| **114** | or/111-113 | 8159 |
| **115** | limit 114 to (english language and humans) | 5945 |
| **116** | 110 or 115 | 12979 |
| **117** | limit 116 to yr=”2010 –Current” | 4818 |

CINAHL via EBSCO. This search was conducted on 07/03/2013.

| **No.** | **Search Terms** | **Results** |
| --- | --- | --- |
| **S1** | (MH "Physical Examination+") | 53,704 |
| **S2** | (MH "Hip") or (MH "Hip Injuries+") or (MH "Hip Joint") | 9314 |
| **S3** | S1 and S2 | 649 |
| **S4** | (clinical n2 exam*) or (clinical n2 sign*) or (clinical n2 test*) or (clinical n2 evaluation) or (clinical n2 investigation) or (clinical n2 diagnosis) or (clinical n2 analysis) or (physical n2 exam*) or (physical n2 sign*) or (physical n2 test*) or (physical n2 evaluation) or (physical n2 investigation) or (physical n2 diagnosis) or (physical n2 analysis) | 57390 |
| **S5** | objective n2 exam* | 9135 |
| **S6** | special n2 test* | 175 |
| **S7** | S4 or S5 or S6 | 65688 |
| **S8** | hip or hips or hip joint or pelvis or groin or femur or femoral or acetabul* or trochanter or iliofemoral or ischiofemoral or pubofemoral or iliopsoas or sartorius or quadriceps or rectus femoris or tensor fascia lat?e or pectineus or adductor brevis or adductor longus or adductor magnus or gluteus medius or gluteus minimus or gluteus maximus or glute* or hamstrings or adductors or adductor magnus or adductor longus or adductor brevis or gracilis or obturator internus or obturator externus or gemell* or quadratus femoris or piriformis or semitendinosus or semimembranosus or biceps femoris or iliacus or obturator or iliotibial | 35601 |
| **S9** | S7 and S8 | 2576 |

After Limiting S3 and S9 “English”, “Humans” and “June 2010 to March 2013”, a total of 353 articles were left.
