# Supplementary material for: A systematic review of the diagnostic performance of orthopedic physical examination tests of the hip
Source: BMC Musculoskelet Disord. 2013 Aug 30;14:257. doi: 10.1186/1471-2474-14-257 (PMC3766647; doi:10.1186/1471-2474-14-257)
Supplement: Additional file 2 — Diagnostic performances of physical test-hip pathology combinations included in review. File is a table of diagnostic characteristics of physical test-hip pathology combinations (sensitivity, specificity, positive and negative predictive values, and positive and negative likelihood ratios) from studies included in this review. [file 1471-2474-14-257-S2.docx]

**Additional file 2. Diagnostic performances of physical test-hip pathology combinations included in review**

**Article Title:** A systematic review of the diagnostic performance of orthopedic physical examination tests of the hip.

**Journal**: BMC Musculoskeletal Disorders

**Authors**: Labib A. Rahman^1^; Sam Adie^1,2,3^ ; Justine M. Naylor ^1,2,3^; Rajat Mittal^1,2,3^; Sarah So^1^; Ian A. Harris^1,2,3^

^1^South West Sydney Clinical School, University of New South Wales, ^2^Orthopaedic Department, Liverpool Hospital, ^3^Whitlam Orthopaedic Research Centre

| **Study** | **Test** | **Pathology** | **Reference Standard** | **Sensitivity** | **Specificity** | **PPV** | **NPV** | **+LR** | **-LR** |  |
| --- | --- | --- | --- | --- | --- | --- | --- | --- | --- | --- |
|  |  |  |  | **(95%CI)** | **(95%CI)** |  |  | **(95%CI)** | **(95%CI)** |  |
|  |  |  |  | **TP/ (TP+FN)** | **TN/ (TN+FP)** |  |  |  |  |  |
| **Adams et al. 1997 [1]** | Patellar-Pubic Percussion | Traumatic Fracture | Radiography | 0.79 | 0.95 | 0.94 | 0.84 | 17.37 | 0.22 |  |
|  |  |  |  | 0.65-0.83 | 0.84-0.99 |  |  | 3.97-98.43 | 0.17-0.42 |  |
|  |  |  |  | 15/19 | 21/22 |  |  |  |  |  |
| **Anwar et al. 1993 [2]** | Trendelenburg Sign | Greater Trochanter Nonunion following Cemented THA in patients with neglected Congenital Dislocation of the Hip (CDH) | Radiography (implied) | 0.80 | 0.71 | 0.53 | 0.89 | 2.74 | 0.28 |  |
|  |  |  |  | 0.54-0.94 | 0.60-0.77 |  |  | 1.36-4.02 | 0.08-0.76 |  |
|  |  |  |  | 8/10 | 17/24 |  |  |  |  |  |
| **Bache et al. 1984 [3]** | Bartford test | Fractured neck of femur | Radiography | 0.91 | 0.82 | 0.86 | 0.88 | 5.01 | 0.11 |  |
|  |  |  |  | 083 – 0.96 | 0.72 – 0.88 |  |  | 2.92 – 8.20 | 0.04 – 0.28 |  |
|  |  |  |  | 51/56 | 36/44 |  |  |  |  |  |
| **Bird et al. 2001 [4]** | Trendelenburg Sign | Partial or Complete Gluteus Medius Tendon Tear | MRI | 0.73 | 0.77 | 0.73 | 0.77 | 3.15 | 0.35 |  |
|  |  |  |  | 0.51-0.87 | 0.59-0.89 |  |  | 1.24-7.84 | 0.15-0.83 |  |
|  |  |  |  | 8/11 | 10/13 |  |  |  |  |  |
| **Bird et al. 2001 [4]** | Pain on Resisted Abduction | Partial or Complete Gluteus Medius Tendon Tear | MRI | 0.73 | 0.46 | 0.53 | 0.67 | 1.35 | 0.59 |  |
|  |  |  |  | 0.52-0.89 | 0.29-0.60 |  |  | 0.73-2.21 | 0.19-1.68 |  |
|  |  |  |  | 8/11 | 6/13 |  |  |  |  |  |
| **Bird et al. 2001 [4]** | Pain on Resisted Adduction | Partial or Complete Gluteus Medius Tendon Tear | MRI | 0.55 | 0.69 | 0.60 | 0.64 | 1.77 | 0.66 |  |
|  |  |  |  | 0.34-0.72 | 0.51-0.84 |  |  | 0.69-4.54 | 0.33-1.29 |  |
|  |  |  |  | 6/11 | 9/13 |  |  |  |  |  |
| **Birrell et al. 2001 [5]** | Restriction in One Plane from Either: (1) Passive Flexion ROM < 94^o^, (2) External Rotation ROM < 23^o^ or (3) Internal Rotation ROM < 23^o^. Measured in seated position with fluid plurimeter. | Mild-Moderate Symptomatic Osteoarthritis | Radiography | 0.86 | 0.54 | 0.60 | 0.83 | 1.85 | 0.27 |  |
|  |  |  |  | 0.79-0.91 | 0.48-0.58 |  |  | 1.52-2.17 | 0.15-0.44 |  |
|  |  |  |  | 72/84 | 57/106 |  |  |  |  |  |
| **Birrell et al. 2001 [5]** | Restriction in Two Planes from Either: (1) Passive Flexion ROM < 94^o^, (2) External Rotation ROM < 23^o^ or (3) Internal Rotation ROM < 23^o^. Measured in seated position with fluid plurimeter. | Mild-Moderate Symptomatic Osteoarthritis | Radiography | 0.56 | 0.77 | 0.66 | 0.69 | 2.47 | 0.57 |  |
|  |  |  |  | 0.48-0.63 | 0.71-0.83 |  |  | 1.69-3.64 | 0.45-0.72 |  |
|  |  |  |  | 47/84 | 82/106 |  |  |  |  |  |
| **Birrell et al. 2001 [5]** | Restriction in Three Planes: (1) Passive Flexion ROM < 94^o^, (2) External Rotation ROM < 23^o^ and (3) Internal Rotation ROM < 23^o^. Measured in seated position with fluid plurimeter. | Mild-Moderate Symptomatic Osteoarthritis | Radiography | 0.32 | 0.93 | 0.79 | 0.63 | 4.87 | 0.73 |  |
|  |  |  |  | 0.26-0.36 | 0.89-0.97 |  |  | 2.32-10.57 | 0.66-0.83 |  |
|  |  |  |  | 27/84 | 99/106 |  |  |  |  |  |
| **Garcia et al. 2010 [6]** | Trendelenburg Sign | Abductor Avulsion Following Primary THA | Sonography | 0.57 | 0.85 | 0.50 | 0.88 | 3.86 | 0.50 |  |
|  |  |  |  | 0.28-0.81 | 0.78-0.91 |  |  | 1.25 – 9.34 | 0.21-0.93 |  |
|  |  |  |  | 4/7 | 23/27 |  |  |  |  |  |
| **Hananouchi et al. 2012 [7]** | Anterior impingement test | Anterosuperior labral lesions | MRI | 0.51 | 0.89 | 0.96 | 0.27 | 4.55 | 0.56 |  |
|  |  |  |  | 0.46 – 0.52 | 0.66 – 0.98 |  |  | 1.35 – 26.64 | 0.49 – 0.82 |  |
|  |  |  |  | 45/89 | 16/18 |  |  |  |  |  |
| **Holla et al. 2012 [8]** | Assisted active hip flexion < 114^o^ as measured with goniometer | Early symptomatic hip osteoarthritis | Radiography | 0.58 | 0.66 | 0.36 | 0.82 | 1.69 | 0.64 |  |
|  |  |  |  | 0.47–0.68 | 0.59–0.72 |  |  | 1.30–2.20 | 0.48–0.85 |  |
|  |  |  |  | 44/76 | 150/228 |  |  |  |  |  |
| **Holla et al. 2012 [8]** | Assisted active hip internal rotation < 24^o^ as measured with goniometer | Early symptomatic hip osteoarthritis | Radiography | 0.56 | 0.78 | 0.46 | 0.84 | 2.55 | 0.56 |  |
|  |  |  |  | 0.45–0.67 | 0.72–0.83 |  |  | 1.86–3.51 | 0.43–0.73 |  |
|  |  |  |  | 42/75 | 178/228 |  |  |  |  |  |
| **Hossain et al. 2007 [9]** | Restricted Straight Leg Raise | Radiologically Occult Hip Fracture | MRI | 0.50 | 0.42 | 0.42 | 0.50 | 0.86 | 1.19 |  |
|  |  |  |  | 0.37-0.64 | 0.31-0.54 |  |  | 0.53-1.38 | 0.68-2.04 |  |
|  |  |  |  | 13/26 | 13/31 |  |  |  |  |  |
| **Hossain et al. 2007 [9]** | Log Roll Test (Passive Rotation) | Radiologically Occult Hip Fracture | MRI | 0.62 | 0.48 | 0.50 | 0.60 | 1.19 | 0.79 |  |
|  |  |  |  | 0.48-0.75 | 0.37-0.59 |  |  | 0.75-1.83 | 0.43-1.43 |  |
|  |  |  |  | 16/26 | 15/31 |  |  |  |  |  |
| **Hossain et al. 2007 [9]** | Pain on Axial Loading | Radiologically Occult Hip Fracture | MRI | 0.73 | 0.58 | 0.59 | 0.72 | 1.74 | 0.46 |  |
|  |  |  |  | 0.59-0.84 | 0.46-0.68 |  |  | 1.10-2.61 | 0.23-0.88 |  |
|  |  |  |  | 19/26 | 18/31 |  |  |  |  |  |
| **Khadilkar et al. 2001 [10]^a^** | Hip Abduction Sign^b^ | Sarcoglycan-opathies in patients with known muscular dystrophy | Immunocyto-chemistry | 0.76 | 0.98 | 0.89 | 0.95 | 34.29 | 0.24 |  |
|  |  |  |  | 0.61-0.83 | 0.94-0.99 |  |  | 10.97 – 122.30 | 0.17-0.41 |  |
|  |  |  |  | 16/21 | 88/90 |  |  |  |  |  |
| **Narvani et al. 2003 [11]** | Discomfort on Internal Rotation-Flexion-Axial Compression Manoeuvre | Acetabular Labral Tears | MRA | 0.75 | 0.43 | 0.27 | 0.86 | 1.31 | 0.58 |  |
|  |  |  |  | 0.34-0.95 | 0.31-0.49 |  |  | 0.50-1.86 | 0.10-2.11 |  |
|  |  |  |  | 3/4 | 6/14 |  |  |  |  |  |
| **Olsson et al. 1981 [12]** | Trendelenburg Sign | Cemented Femoral Stem Loosening Post-THA | Radiography | 0.47 | 0.81 | 0.56 | 0.75 | 2.50 | 0.66 |  |
|  |  |  |  | 0.33-0.59 | 0.75-0.88 |  |  | 1.31-4.70 | 0.47-0.89 |  |
|  |  |  |  | 14/30 | 48/59 |  |  |  |  |  |
| **Olsson et al. 1981 [12]** | Painful or Impossible Active Straight Leg-Raise | Cemented Femoral Stem Loosening Post-THA | Radiography | 0.19 | 0.93 | 0.50 | 0.71 | 2.65 | 0.90 |  |
|  |  |  |  | 0.10-0.29 | 0.89-0.96 |  |  | 0.87-8.04 | 0.74-1.02 |  |
|  |  |  |  | 5/26 | 64/69 |  |  |  |  |  |
| **Röder et al. 2003 [13]** | Pain on Axial Compression | Uncemented Acetabular Cup Loosening Post-THA | Radiography | 0.00 | 1.00 | 0.00 | 0.99 | 7.23 | 0.98 |  |
|  |  |  |  | 0.00-0.13 | 1.00-1.00 |  |  | 0.71 – 67.39 | 0.84 – 1.00 |  |
|  |  |  |  | 0/17 | 1685/ 1691 |  |  |  |  |  |
| **Röder et al. 2003 [13]** | Pain on Axial Compression | Uncemented Acetabular Cup Loosening Post-THA | Radiography | 0.13 | 0.99 | 0.29 | 0.96 | 8.63 | 0.89 |  |
|  |  |  |  | 0.05-0.23 | 0.98-0.99 |  |  | 2.93-24.30 | 0.78-0.97 |  |
|  |  |  |  | 4/32 | 680/690 |  |  |  |  |  |
| **Pooled Data: Röder et al. 2003 [13]** | Pain on Axial Compression | Uncemented Acetabular Cup Loosening Post-THA | Radiography | 0.08 | 0.99 | 0.20 | 0.98 | 12.15 | 0.93 |  |
|  |  |  |  | 0.03-0.17 | 0.99-1.00 |  |  | 4.33 – 32.83 | 0.84 – 0.97 |  |
|  |  |  |  | 4/49 | 2365/ 2381 |  |  |  |  |  |
| **Röder et al. 2003 [13]** | Pain on Internal Rotation | Uncemented Acetabular Cup Loosening Post-THA | Radiography | 0.19 | 0.97 | 0.08 | 0.99 | 6.69 | 0.83 |  |
|  |  |  |  | 0.08-0.39 | 0.97-0.97 |  |  | 2.60-14.91 | 0.63-0.95 |  |
|  |  |  |  | 4/21 | 1639/ 1687 |  |  |  |  |  |
| **Röder et al. 2003 [13]** | Pain on Internal Rotation | Uncemented Acetabular Cup Loosening Post-THA | Radiography | 0.21 | 0.96 | 0.19 | 0.96 | 4.72 | 0.83 |  |
|  |  |  |  | 0.11-0.35 | 0.95-0.96 |  |  | 2.21-9.40 | 0.68-0.94 |  |
|  |  |  |  | 7/34 | 658/688 |  |  |  |  |  |
| **Pooled Data: Röder et al. 2003 [13]** | Pain on Internal Rotation | Uncemented Acetabular Cup Loosening Post-THA | Radiography | 0.20 | 0.97 | 0.12 | 0.07 | 6.09 | 0.83 |  |
|  |  |  |  | 0.12 – 0.31 | 0.97 – 0.97 |  |  | 3.39 – 10.37 | 0.71 – 0.91 |  |
|  |  |  |  | 11/ 55 | 2297/ 2375 |  |  |  |  |  |
| **Röder et al. 2003 [13]** | Pain on External Rotation | Uncemented Acetabular Cup Loosening Post-THA | Radiography | 0.06 | 0.99 | 0.07 | 0.99 | 7.22 | 0.95 |  |
|  |  |  |  | 0.01-0.22 | 0.99-0.99 |  |  | 1.22-37.83 | 0.78-1.00 |  |
|  |  |  |  | 1/18 | 1677/ 1690 |  |  |  |  |  |
| **Röder et al. 2003 [13]** | Pain on External Rotation | Uncemented Acetabular Cup Loosening Post-THA | Radiography | 0.06 | 0.99 | 0.25 | 0.96 | 7.43 | 0.94 |  |
|  |  |  |  | 0.02-0.15 | 0.99-1.00 |  |  | 1.74-30.74 | 0.86-0.99 |  |
|  |  |  |  | 2/31 | 685/ 691 |  |  |  |  |  |
| **Pooled Data: Röder et al. 2003 [13]** | Pain on External Rotation | Uncemented Acetabular Cup Loosening Post-THA | Radiography | 0.06 | 0.99 | 0.14 | 0.98 | 7.67 | 0.95 |  |
|  |  |  |  | 0.02 – 0.14 | 0.99 – 0.99 |  |  | 2.45 – 22.97 | 0.86 – 0.99 |  |
|  |  |  |  | 3/ 49 | 2362/ 2381 |  |  |  |  |  |
| **Röder et al. 2003 [13]** | Pain on Axial Compression | Cemented Acetabular Cup Loosening Post-THA | Radiography | 0.00 | 0.99 | 0.00 | 0.99 | 6.44 | 0.95 |  |
|  |  |  |  | 0.00-0.26 | 0.99-1.00 |  |  | 0.64 – 54.06 | 0.63 – 77.34 |  |
|  |  |  |  | 0/8 | 746/ 752 |  |  |  |  |  |
| **Röder et al. 2003 [13]** | Pain on Axial Compression | Cemented Acetabular Cup Loosening Post-THA | Radiography | 0.02 | 1.00 | 0.33 | 0.88 | 3.63 | 0.99 |  |
|  |  |  |  | 0.00-0.04 | 0.99-1.00 |  |  | 0.48-27.51 | 0.96-1.00 |  |
|  |  |  |  | 1/61 | 441/443 |  |  |  |  |  |
| **Pooled Data: Röder et al. 2003 [13]** | Pain on Axial Compression | Cemented Acetabular Cup Loosening Post-THA | Radiography | 0.01 | 0.99 | 0.11 | 0.95 | 2.17 | 0.99 |  |
|  |  |  |  | 0.00 – 0.06 | 0.99 – 1.00 |  |  | 0.35 – 13.00 | 0.95 – 1.01 |  |
|  |  |  |  | 1/ 69 | 1187/ 1195 |  |  |  |  |  |
| **Röder et al. 2003 [13]** | Pain on Internal Rotation | Cemented Acetabular Cup Loosening Post-THA | Radiography | 1.00 | 0.95 | 0.05 | 1.00 | 16.87 | 0.18 |  |
|  |  |  |  | 0.35-1.00 | 0.95-0.95 |  |  | 6.08 – 20.12 | 0.02 – 0.72 |  |
|  |  |  |  | 2/2 | 721/ 758 |  |  |  |  |  |
| **Röder et al. 2003 [13]** | Pain on Internal Rotation | Cemented Acetabular Cup Loosening Post-THA | Radiography | 0.21 | 0.93 | 0.27 | 0.90 | 2.80 | 0.86 |  |
|  |  |  |  | 0.13-0.30 | 0.92-0.94 |  |  | 1.52-4.96 | 0.74-0.95 |  |
|  |  |  |  | 12/58 | 413/ 446 |  |  |  |  |  |
| **Pooled Data: Röder et al. 2003 [13]** | Pain on Internal Rotation | Cemented Acetabular Cup Loosening Post-THA | Radiography | 0.23 | 0.94 | 0.17 | 0.96 | 4.01 | 0.81 |  |
|  |  |  |  | 0.15 – 0.34 | 0.94 – 0.95 |  |  | 2.37 – 6.48 | 0.70 – 0.91 |  |
|  |  |  |  | 14/ 60 | 1134/ 1204 |  |  |  |  |  |
| **Röder et al. 2003 [13]** | Pain on External Rotation | Cemented Acetabular Cup Loosening Post-THA | Radiography | 0.02 | 1.00 | 0.50 | 0.88 | 7.54 | 0.99 |  |
|  |  |  |  | 0.00-0.03 | 1.00-1.00 |  |  | 0.79-72.05 | 0.97-1.00 |  |
|  |  |  |  | 1/59 | 444/445 |  |  |  |  |  |
| **Röder et al. 2003 [13]** | Pain on External Rotation | Cemented Acetabular Cup Loosening Post-THA | Radiography | 0.00 | 1.00 | 0.00 | 0.99 | 16.73 | 0.95 |  |
|  |  |  |  | 0.00-0.16 | 1.00-1.00 |  |  | 1.51 – 172.69 | 0.78 – 1.00 |  |
|  |  |  |  | 0/8 | 750/ 752 |  |  |  |  |  |
| **Pooled Data: Röder et al. 2003 [13]** | Pain on External Rotation | Cemented Acetabular Cup Loosening Post-THA | Radiography | 0.02 | 1.00 | 0.25 | 0.95 | 5.96 | 0.99 |  |
|  |  |  |  | 0.00 – 0.04 | 1.00 – 1.00 |  |  | 0.86 – 41.13 | 0.96 – 1.00 |  |
|  |  |  |  | 1/67 | 1194 /1197 |  |  |  |  |  |
| **Röder et al. 2003 [13]** | Pain on Axial Compression | Uncemented Femoral Stem Loosening Post-THA | Radiography | 0.08 | 0.99 | 0.33 | 0.94 | 7.00 | 0.93 |  |
|  |  |  |  | 0.08-0.02 | 0.98-1.00 |  |  | 0.93-51.26 | 0.81-1.00 |  |
|  |  |  |  | 1/12 | 166/168 |  |  |  |  |  |
| **Röder et al. 2003 [13]** | Pain on Axial Compression | Uncemented Femoral Stem Loosening Post-THA | Radiography | 0.05 | 0.99 | 0.20 | 0.96 | 5.88 | 0.96 |  |
|  |  |  |  | 0.01-0.16 | 0.99-1.00 |  |  | 0.89-37.05 | 0.84-1.00 |  |
|  |  |  |  | 1/19 | 443/ 447 |  |  |  |  |  |
| **Pooled Data: Röder et al. 2003 [13]** | Pain on Axial Compression | Uncemented Femoral Stem Loosening Post-THA | Radiography | 0.07 | 0.99 | 0.25 | 0.96 | 6.61 | 0.95 |  |
|  |  |  |  | 0.02 – 0.15 | 0.99 – 1.00 |  |  | 1.55 – 27.35 | 0.86 – 0.99 |  |
|  |  |  |  | 2/ 31 | 609 /615 |  |  |  |  |  |
| **Röder et al. 2003 [13]** | Pain on Internal Rotation | Uncemented Femoral Stem Loosening Post-THA | Radiography | 0.18 | 0.96 | 0.17 | 0.96 | 4.25 | 0.86 |  |
|  |  |  |  | 0.08-0.35 | 0.95-0.97 |  |  | 1.57-10.35 | 0.67-0.97 |  |
|  |  |  |  | 4/22 | 425/ 444 |  |  |  |  |  |
| **Röder et al. 2003 [13]** | Pain on Internal Rotation | Uncemented Femoral Stem Loosening Post-THA | Radiography | 0.33 | 0.93 | 0.27 | 0.95 | 5.09 | 0.71 |  |
|  |  |  |  | 0.15-0.57 | 0.92-0.95 |  |  | 1.84-11.80 | 0.45-0.93 |  |
|  |  |  |  | 4/12 | 157/ 168 |  |  |  |  |  |
| **Pooled Data: Röder et al. 2003 [13]** | Pain on Internal Rotation | Uncemented Femoral Stem Loosening Post-THA | Radiography | 0.24 | 0.95 | 0.21 | 0.96 | 4.80 | 0.80 |  |
|  |  |  |  | 0.13 – 0.38 | 0.95 – 0.96 |  |  | 2.35 – 9.14 | 0.65 – 0.92 |  |
|  |  |  |  | 8 /34 | 582 /612 |  |  |  |  |  |
| **Röder et al. 2003 [13]** | Pain on External Rotation | Uncemented Femoral Stem Loosening Post-THA | Radiography | 0.00 | 0.99 | 0.00 | 0.93 | 2.60 | 0.98 |  |
|  |  |  |  | 0.00-0.11 | 0.99-1.00 |  |  | 0.23 – 27.49 | 0.85 – 1.01 |  |
|  |  |  |  | 0/12 | 166/ 168 |  |  |  |  |  |
| **Röder et al. 2003 [13]** | Pain on External Rotation | Uncemented Femoral Stem Loosening Post-THA | Radiography | 0.05 () | 0.99 () | 0.14 | 0.96 | 3.92 | 0.96 |  |
|  |  |  |  | 0.01-0.18 | 0.99-0.99 |  |  | 0.62-22.91 | 0.83-1.01 |  |
|  |  |  |  | 1/19 | 441/ 447 |  |  |  |  |  |
| **Pooled Data: Röder et al. 2003 [13]** | Pain on External Rotation | Uncemented Femoral Stem Loosening Post-THA | Radiography | 0.03 | 0.99 | 0.11 | 0.95 | 2.48 | 0.98 |  |
|  |  |  |  | 0.01 – 0.12 | 0.99 – 0.99 |  |  | 0.40 – 14.42 | 0.89 – 1.01 |  |
|  |  |  |  | 1 /31 | 607 /615 |  |  |  |  |  |
| **Röder et al. 2003 [13]** | Pain on Axial Compression | Cemented Femoral Stem Loosening Post-THA | Radiography | 0.02 | 0.99 | 0.14 | 0.87 | 1.12 | 1.00 |  |
|  |  |  |  | 0.00-0.04 | 0.99-0.99 |  |  | 0.28-4.37 | 0.97-1.01 |  |
|  |  |  |  | 2/136 | 898/ 910 |  |  |  |  |  |
| **Röder et al. 2003 [13]** | Pain on Axial Compression | Cemented Femoral Stem Loosening Post-THA | Radiography | 0.04 | 1.00 | 0.43 | 0.96 | 17.34 | 0.97 |  |
|  |  |  |  | 0.01-0.06 | 1.00-1.00 |  |  | 4.38-68.47 | 0.94-0.99 |  |
|  |  |  |  | 3/83 | 1915/ 1919 |  |  |  |  |  |
| **Pooled Data: Röder et al. 2003 [13]** | Pain on Axial Compression | Cemented Femoral Stem Loosening Post-THA | Radiography | 0.02 | 0.99 | 0.24 | 0.93 | 4.04 | 0.98 |  |
|  |  |  |  | 0.01 – 0.04 | 0.99 – 1.00 |  |  | 1.54 – 10.48 | 0.96 – 1.00 |  |
|  |  |  |  | 5 /219 | 2813/ 2829 |  |  |  |  |  |
| **Röder et al. 2003 [13]** | Pain on Internal Rotation | Cemented Femoral Stem Loosening Post-THA | Radiography | 0.15 | 0.95 | 0.30 | 0.88 | 2.82 | 0.90 |  |
|  |  |  |  | 0.10-0.20 | 0.94-0.96 |  |  | 1.72-4.56 | 0.84-0.96 |  |
|  |  |  |  | 20/137 | 862/ 909 |  |  |  |  |  |
| **Röder et al. 2003 [13]** | Pain on Internal Rotation | Cemented Femoral Stem Loosening Post-THA | Radiography | 0.17 | 0.97 | 0.21 | 0.97 | 5.96 | 0.86 |  |
|  |  |  |  | 0.10-0.26 | 0.97-0.98 |  |  | 3.31-10.34 | 0.76-0.93 |  |
|  |  |  |  | 12/72 | 1876/ 1930 |  |  |  |  |  |
| **Pooled Data: Röder et al. 2003 [13]** | Pain on Internal Rotation | Cemented Femoral Stem Loosening Post-THA | Radiography | 0.15 | 0.96 | 0.24 | 0.94 | 4.30 | 0.88 |  |
|  |  |  |  | 0.11 – 0.20 | 0.96 – 0.97 |  |  | 2.96 – 6.19 | 0.83 – 0.92 |  |
|  |  |  |  | 32/ 209 | 2738 /2839 |  |  |  |  |  |
| **Röder et al. 2003 [13]** | Pain on External Rotation | Cemented Femoral Stem Loosening Post-THA | Radiography | 0.02 | 0.99 | 0.25 | 0.87 | 2.21 | 0.99 |  |
|  |  |  |  | 0.00-0.03 | 0.99-1.00 |  |  | 0.51-9.48 | 0.97-1.00 |  |
|  |  |  |  | 2/137 | 903/ 909 |  |  |  |  |  |
| **Röder et al. 2003 [13]** | Pain on External Rotation | Cemented Femoral Stem Loosening Post-THA | Radiography | 0.06 | 1.00 | 0.56 | 0.96 | 28.19 | 0.94 |  |
|  |  |  |  | 0.03-0.09 | 1.00-1.00 |  |  | 8.30-95.94 | 0.92-0.98 |  |
|  |  |  |  | 5/85 | 1913/ 1917 |  |  |  |  |  |
| **Pooled Data: Röder et al. 2003 [13]** | Pain on External Rotation | Cemented Femoral Stem Loosening Post-THA | Radiography | 0.03 | 1.00 | 0.41 | 0.22 | 8.91 | 0.97 |  |
|  |  |  |  | 0.02 – 0.05 | 1.00 – 1.00 |  |  | 3.53 – 22.43 | 0.95 – 0.99 |  |
| **Röder et al. 2003 [13]** | Flexion ROM < 70^o^ | Uncemented Acetabular Cup Loosening | Radiography | 0.07 | 0.98 | 0.12 | 0.96 | 3.07 | 0.95 | |
|  |  |  |  | 0.02-0.18 | 0.98-0.98 |  |  | 0.79-11.05 | 0.83-1.01 | |
|  |  |  |  | 2/30 | 675/ 690 |  |  |  |  |  |
| **Röder et al. 2003 [13]** | Flexion ROM < 70^o^ | Uncemented Acetabular Cup Loosening | Radiography | 0.00 | 0.98 | 0.00 | 0.98 | 0.83 | 1.00 | |
|  |  |  |  | 0.00-0.10 | 0.98-0.99 |  |  | 0.09 – 7.41 | 0.90 – 1.02 | |
|  |  |  |  | 0/33 | 1641/ 1670 |  |  |  |  |  |
| **Pooled Data: Röder et al. 2003 [13]** | Flexion ROM < 70^o^ | Uncemented Acetabular Cup Loosening | Radiography | 0.06 | 0.98 | 0.04 | 0.99 | 3.25 | 0.96 | |
|  |  |  |  | 0.01-0.21 | 0.98-0.98 |  |  | 0.55-12.31 | 0.81-1.01 | |
|  |  |  |  | 2/32 | 2316/2360 |  |  |  |  |  |
| **Röder et al. 2003 [13]** | Flexion ROM < 70^o^ | Cemented Acetabular Cup Loosening | Radiography | 0.00 | 0.96 | 0.00 | 0.99 | 1.39 | 0.98 | |
|  |  |  |  | 0.00-0.34 | 0.96-0.96 |  |  | 0.14 – 9.43 | 0.64 – 1.04 | |
|  |  |  |  | 0/7 | 712/ 745 |  |  |  |  |  |
| **Röder et al. 2003** | Flexion ROM < 70^o^ | Cemented Acetabular Cup Loosening | Radiography | 0.08 | 0.94 | 0.17 | 0.88 | 1.43 | 0.97 | |
|  |  |  |  | 0.04-0.16 | 0.94-0.95 |  |  | 0.57-3.41 | 0.88-1.03 | |
|  |  |  |  | 5/61 | 410/ 435 |  |  |  |  |  |
| **Pooled Data: Röder et al. 2003 [13]** | Flexion ROM < 70^o^ | Cemented Acetabular Cup Loosening | Radiography | 0.07 | 0.95 | 0.08 | 0.95 | 1.50 | 0.97 | |
|  |  |  |  | 0.03-0.16 | 0.95-0.96 |  |  | 0.54-3.65 | 0.88-1.03 | |
|  |  |  |  | 5/68 | 1122/1180 |  |  |  |  |  |
| **Röder et al. 2003 [13]** | Flexion ROM < 70^o^ | Uncemented Femoral Stem Loosening | Radiography | 0.14 | 0.97 | 0.18 | 0.96 | 4.51 | 0.89 | |
|  |  |  |  | 0.05-0.31 | 0.96-0.98 |  |  | 1.43-12.90 | 0.71-0.98 | |
|  |  |  |  | 3/21 | 428/ 442 |  |  |  |  |  |
| **Röder et al. 2003 [13]** | Flexion ROM < 70^o^ | Uncemented Femoral Stem Loosening | Radiography | 0.15 | 0.99 | 0.67 | 0.94 | 25.69 | 0.85 | |
|  |  |  |  | 0.05-0.22 | 0.99-1.00 |  |  | 3.48-193.36 | 0.78-0.96 | |
|  |  |  |  | 2/13 | 166/ 167 |  |  |  |  |  |
| **Pooled Data: Röder et al. 2003 [13]** | Flexion ROM < 70^o^ | Uncemented Femoral Stem Loosening | Radiography | 0.15 | 0.98 | 0.25 | 0.95 | 5.97 | 0.87 | |
|  |  |  |  | 0.06-0.28 | 0.97-0.98 |  |  | 1.95-16.128 | 0.73-0.97 | |
|  |  |  |  | 5/34 | 594/609 |  |  |  |  |  |
| **Röder et al. 2003 [13]** | Flexion ROM < 70^o^ | Cemented Femoral Stem Loosening | Radiography | 0.03 | 0.95 | 0.04 | 0.96 | 0.51 | 1.03 | |
|  |  |  |  | 0.01-0.08 | 0.95-0.95 |  |  | 0.14-1.81 | 0.96-1.05 | |
|  |  |  |  | 2/80 | 1819/ 1912 |  |  |  |  |  |
| **Röder et al. 2003 [13]** | Flexion ROM < 70^o^ | Cemented Femoral Stem Loosening | Radiography | 0.07 | 0.96 | 0.20 | 0.87 | 1.67 | 0.97 | |
|  |  |  |  | 0.04-0.11 | 0.96-0.97 |  |  | 0.83-3.33 | 0.92-1.01 | |
|  |  |  |  | 9/138 | 863/ 898 |  |  |  |  |  |
| **Pooled Data: Röder et al. 2003 [13]** | Flexion ROM < 70^o^ | Cemented Femoral Stem Loosening | Radiography | 0.05 | 0.95 | 0.08 | 0.93 | 1.11 | 1.00 | |
|  |  |  |  | 0.03-0.09 | 0.95-0.96 |  |  | 0.57-2.06 | 0.95-1.02 | |
|  |  |  |  | 11/218 | 2682/2810 |  |  |  |  |  |
| **Shin et al. 1996 [14]** | Active Flexion ROM < 113^o^. Measured using Goniometer. | Femoral Neck Stress Fracture (radiologically occult but suggestive bone scintigraphy) | 6-week Follow up Radiography | 0.00 | 1.00 | - | 0.32 | 0.50 | 1.04 |  |
|  |  |  |  | 0.00-0.00 | 1.00-1.00 |  |  | 0.03 – 8.57 | 0.94 – 1.15 |  |
|  |  |  |  | 0/13 | 6/6 |  |  |  |  |  |
| **Shin et al. 1996 [14]** | Active Extension ROM < 28^o^. Measured using Goniometer. | Femoral Neck Stress Fracture (radiologically occult but suggestive bone scintigraphy) | 6-week Follow up Radiography | 0.69 | 0.67 | 0.82 | 0.50 | 2.08 | 0.46 |  |
|  |  |  |  | 0.55-0.80 | 0.35-0.89 |  |  | 0.84-7.16 | 0.23-1.31 |  |
|  |  |  |  | 9/13 | 4/6 |  |  |  |  |  |
| **Shin et al. 1996 [14]** | Active Internal Rotation ROM < 45^o^. Measured using Goniometer. | Femoral Neck Stress Fracture (radiologically occult but suggestive bone scintigraphy) | 6-week Follow up Radiography | 0.77 | 0.00 | 0.63 | 0.00 | 0.81 | 3.50 |  |
|  |  |  |  | 0.77-0.89 | 0.00-0.26 |  |  | 0.72 – 1.28 | 0.40 – 37.01 |  |
|  |  |  |  | 10/13 | 0/6 |  |  |  |  |  |
| **Shin et al. 1996 [14]** | Active External Rotation ROM < 45^o^. Measured using Goniometer. | Femoral Neck Stress Fracture (radiologically occult but suggestive bone scintigraphy) | 6-week Follow up Radiography | 0.15 | 0.50 | 0.40 | 0.21 | 0.31 | 1.69 |  |
|  |  |  |  | 0.06-0.28 | 0.29-0.78 |  |  | 0.08-1.29 | 0.92-3.32 |  |
|  |  |  |  | 2/13 | 3/6 |  |  |  |  |  |
| **Shin et al. 1996 [14]** | Active Abduction ROM < 48^o^. Measured using Goniometer. | Femoral Neck Stress Fracture (radiologically occult but suggestive bone scintigraphy) | 6-week Follow up Radiography | 0.62 | 0.33 | 0.67 | 0.29 | 0.92 | 1.15 |  |
|  |  |  |  | 0.51-0.76 | 0.11-0.64 |  |  | 0.57-2.10 | 0.38-4.62 |  |
|  |  |  |  | 8/13 | 2/6 |  |  |  |  |  |
| **Shin et al. 1996 [14]** | Pain on Log Roll Test | Femoral Neck Stress Fracture (radiologically occult but suggestive bone scintigraphy) | 6-week Follow up Radiography | 1.00 | 0.33 | 0.76 | 1.00 | 1.50 | 0.10 |  |
|  |  |  |  | 0.90-1.00 | 0.12-0.33 |  |  | 1.00 – 1.72 | 0.01 – 0.98 |  |
|  |  |  |  | 13/13 | 2/6 |  |  |  |  |  |
| **Shin et al. 1996 [14]** | Pain on Active Straight Leg Raise | Femoral Neck Stress Fracture (radiologically occult but suggestive bone scintigraphy) | 6-week Follow up Radiography | 0.92 | 0.17 | 0.71 | 0.50 | 1.11 | 0.46 |  |
|  |  |  |  | 0.86-0.99 | 0.03-0.30 |  |  | 0.89-1.41 | 0.05-4.26 |  |
|  |  |  |  | 12/13 | 1/6 |  |  |  |  |  |
| **Sutlive et al. 2008 [15]** | Lateral Pain on Active Hip Flexion. Patient Supine. | Symptomatic Osteoarthritis | Radiography | 0.43 | 0.88 | 0.60 | 0.79 | 3.64 | 0.65 |  |
|  |  |  |  | 0.28-0.56 | 0.82-0.94 |  |  | 1.52-8.68 | 0.47-0.89 |  |
|  |  |  |  | 9/21 | 45/51 |  |  |  |  |  |
| **Sutlive et al. 2008 [15]** | Pain on Active Hip Extension. Patient Prone. | Symptomatic Osteoarthritis | Radiography | 0.52 | 0.80 | 0.52 | 0.80 | 2.67 | 0.59 |  |
|  |  |  |  | 0.36-0.67 | 0.74-0.87 |  |  | 1.34-5.02 | 0.38-0.88 |  |
|  |  |  |  | 11/21 | 41/51 |  |  |  |  |  |
| **Sutlive et al. 2008 [15]** | Pain on Abduction and/or Adduction. Patient Supine. | Symptomatic Osteoarthritis | Radiography | 0.33 | 0.94 | 0.70 | 0.77 | 5.67 | 0.71 |  |
|  |  |  |  | 0.20-0.42 | 0.89-0.98 |  |  | 1.76-19.05 | 0.59-0.90 |  |
|  |  |  |  | 7/21 | 48/51 |  |  |  |  |  |
| **Sutlive et al. 2008 [15]** | Passive Internal Rotation </= 25^o^. Measured with inclinometer. Patient Prone. | Symptomatic Osteoarthritis | Radiography | 0.76 | 0.61 | 0.44 | 0.86 | 1.94 | 0.39 |  |
|  |  |  |  | 0.58-0.89 | 0.54-0.66 |  |  | 1.25-2.60 | 0.17-0.78 |  |
|  |  |  |  | 16/21 | 31/51 |  |  |  |  |  |
| **Sutlive et al. 2008 [15]** | Squat Test | Symptomatic Osteoarthritis | Radiography | 0.24 | 0.96 | 0.71 | 0.75 | 6.07 | 0.79 |  |
|  |  |  |  | 0.13-0.31 | 0.91-0.99 |  |  | 1.46-26.32 | 0.70-0.96 |  |
|  |  |  |  | 5/21 | 49/51 |  |  |  |  |  |
| **Sutlive et al. 2008 [15]** | Scour Test | Symptomatic Osteoarthritis | Radiography | 0.62 | 0.75 | 0.50 | 0.83 | 2.43 | 0.51 |  |
|  |  |  |  | 0.44-0.77 | 0.67-0.81 |  |  | 1.36-3.97 | 0.29-0.83 |  |
|  |  |  |  | 13/21 | 38/51 |  |  |  |  |  |
| **Sutlive et al. 2008 [15]** | FABER/ Patrick’s Test < 60^o^ | Symptomatic Osteoarthritis | Radiography | 0.57 | 0.71 | 0.44 | 0.80 | 1.94 | 0.61 |  |
|  |  |  |  | 0.40-0.73 | 0.63-0.77 |  |  | 1.08-3.18 | 0.35-0.95 |  |
|  |  |  |  | 12/21 | 36/51 |  |  |  |  |  |
| **Sutlive et al. 2008 [15]** | 5-Part Clinical Prediction Rule ^c^ (>1 Variable positive) | Symptomatic Osteoarthritis | Radiography | 0.95 | 0.18 | 0.32 | 0.90 | 1.16 | 0.27 |  |
|  |  |  |  | 0.82-0.99 | 0.12-0.19 |  |  | 0.94-1.23 | 0.05-1.46 |  |
|  |  |  |  | 20/21 | 9/51 |  |  |  |  |  |
| **Sutlive et al. 2008 [15]** | 5-Part Clinical Prediction Rule ^c^ (>2 Variables Positive) | Symptomatic Osteoarthritis | Radiography | 0.81 | 0.61 | 0.46 | 0.89 | 2.06 | 0.31 |  |
|  |  |  |  | 0.63-0.92 | 0.54-0.65 |  |  | 1.37-2.65 | 0.12-0.68 |  |
|  |  |  |  | 17/21 | 31/51 |  |  |  |  |  |
| **Sutlive et al. 2008 [15]** | 5-Part Clinical Prediction Rule ^c^ (>3 Variables Positive) | Symptomatic Osteoarthritis | Radiography | 0.71 | 0.86 | 0.68 | 0.88 | 5.20 | 0.33 |  |
|  |  |  |  | 0.55-0.84 | 0.79-0.91 |  |  | 2.66-9.57 | 0.18-0.57 |  |
|  |  |  |  | 15/21 | 44/51 |  |  |  |  |  |
| **Sutlive et al. 2008 [15]** | 5-Part Clinical Prediction Rule ^c^ (>4 Variables Positive) | Symptomatic Osteoarthritis | Radiography | 0.48 | 0.98 | 0.91 | 0.82 | 24.29 | 0.53 |  |
|  |  |  |  | 0.34-0.52 | 0.93-1.00 |  |  | 4.64-145.01 | 0.49-0.71 |  |
|  |  |  |  | 10/21 | 50/51 |  |  |  |  |  |
| **Sutlive et al. 2008 [15]** | 5-Part Clinical Prediction Rule ^c^ (All 5 Variables Positive) | Symptomatic Osteoarthritis | Radiography | 0.14 | 0.98 | 0.75 | 0.74 | 7.29 | 0.87 |  |
|  |  |  |  | 0.06-0.18 | 0.95-1.00 |  |  | 1.09-50.33 | 0.82-1.00 |  |
|  |  |  |  | 3/21 | 50/51 |  |  |  |  |  |
| **Tiru et al. 2002 [16]** | Patellar-Pubic Percussion Test^d^ | Traumatic Fracture (Radiologically Occult) | Repeat Radiography, Bone Scintigraphy, MRI or CT | 0.96 | 0.86 | 0.98 | 0.75 | 6.73 | 0.05 |  |
|  |  |  |  | 0.94-0.97 | 0.74-0.93 |  |  | 3.61-14.00 | 0.03-0.08 |  |
|  |  |  |  | 245/ 255 | 30/35 |  |  |  |  |  |

**Table Legend:**

Positive Predictive Value (PPV), Negative Predictive Value (NPV), Positive Likelihood Ratio (+LR), Negative Likelihood Ratio (-LR), 95% Confidence Interval (95%CI), True Positives (TP), False Positives (FP), True Negatives (TN), False Negatives (FN), Range of Motion (ROM). All values rounded to 2 decimal places. When one of the cells of the 2x2 contingency table contained the value ‘zero’, we added 0.5 to each cell in order to calculate likelihood ratio values and their confidence intervals.

^a^10 healthy controls that tested negative with the index test were removed from our calculations

^b^Description of the Hip Abduction Sign in study by Khadilkar and Singh [10]: “*Patients were asked to rise from the ground while they were observed from the front. After the initial efforts to get off the ground, as they began to climb up on their thighs, abduction of hip joints and thighs was noted. The test was considered positive when the angle subtended by both knees was more than 90^o^ (that is, 45^o^ on each side of the midline)”*

^c^Clinical Prediction Rule consisted of 5 variables: (1) self-reported squatting as an aggravating factor, (2) scour test with adduction causing groin or lateral pain, (3) active hip flexion causing late pain, (4) active hip extension causing hip pain, and (5) passive hip internal rotation less than or equal to 25^o^.

^d^Description of the Patellar Pubic Percussion test in the study by Tiru et al. [16]: *“The test was performed by percussing the patella and simultaneously auscultating with the bell of the stethoscope over the pubic symphysis. The percussion note was then compared over the contralateral side in a similar fashion. A positive test was one that resulted in diminished percussion note on the side of pain felt and a negative test was defined as one in which no difference in percussion note was obtained.”*

**References**:

1. Adams SL, Yarnold PR: **Clinical use of the patellar-pubic percussion sign in hip trauma.** *The American journal of emergency medicine* 1997, **15:**173-175.

2. Anwar MM, Sugano N, Masuhara K, Kadowaki T, Takaoka K, Ono K: **Total hip arthroplasty in the neglected congenital dislocation of the hip. A five- to 14-year follow-up study.** *Clinical orthopaedics and related research* 1993**:**127-134.

3. Bache JB, Cross AB: **The Barford test. A useful diagnostic sign in fractures of the femoral neck.** *The Practitioner* 1984, **228:**305-308.

4. Bird PA, Oakley SP, Shnier R, Kirkham BW: **Prospective evaluation of magnetic resonance imaging and physical examination findings in patients with greater trochanteric pain syndrome.** *Arthritis and rheumatism* 2001, **44:**2138-2145.

5. Birrell F, Croft P, Cooper C, Hosie G, Macfarlane G, Silman A: **Predicting radiographic hip osteoarthritis from range of movement.** *Rheumatology (Oxford, England)* 2001, **40:**506-512.

6. Garcia FL, Picado CH, Nogueira-Barbosa MH: **Sonographic evaluation of the abductor mechanism after total hip arthroplasty.** *Journal of ultrasound in medicine : official journal of the American Institute of Ultrasound in Medicine* 2010, **29:**465-471.

7. Hananouchi T, Yasui Y, Yamamoto K, Toritsuka Y, Ohzono K: **Anterior impingement test for labral lesions has high positive predictive value.** *Clin Orthop* 2012, **470:**3524-3529.

8. Holla JF, van der Leeden M, Roorda LD, Bierma-Zeinstra SM, Damen J, Dekker J, Steultjens MP: **Diagnostic accuracy of range of motion measurements in early symptomatic hip and/or knee osteoarthritis.** *Arthritis Care Res (Hoboken)* 2012, **64:**59-65.

9. Hossain M, Barwick C, Sinha AK, Andrew JG: **Is magnetic resonance imaging (MRI) necessary to exclude occult hip fracture?** *Injury* 2007, **38:**1204-1208.

10. Khadilkar SV, Singh RK: **Hip abduction sign: a new clinical sign in sarcoglycanopathies.** *Journal of clinical neuromuscular disease* 2001, **3:**13-15.

11. Narvani AA, Tsiridis E, Kendall S, Chaudhuri R, Thomas P: **A preliminary report on prevalence of acetabular labrum tears in sports patients with groin pain.** *Knee surgery, sports traumatology, arthroscopy : official journal of the ESSKA* 2003, **11:**403-408.

12. Olsson SS, Jernberger A, Tryggo D: **Clinical and radiological long-term results after Charnley-Muller total hip replacement. A 5 to 10 year follow-up study with special reference to aseptic loosening.** *Acta orthopaedica Scandinavica* 1981, **52:**531-542.

13. Roder C, Eggli S, Aebi M, Busato A: **The validity of clinical examination in the diagnosis of loosening of components in total hip arthroplasty.** *The Journal of bone and joint surgery British volume* 2003, **85:**37-44.

14. Shin AY, Morin WD, Gorman JD, Jones SB, Lapinsky AS: **The superiority of magnetic resonance imaging in differentiating the cause of hip pain in endurance athletes.** *The American journal of sports medicine* 1996, **24:**168-176.

15. Sutlive TG, Lopez HP, Schnitker DE, Yawn SE, Halle RJ, Mansfield LT, Boyles RE, Childs JD: **Development of a clinical prediction rule for diagnosing hip osteoarthritis in individuals with unilateral hip pain.** *The Journal of orthopaedic and sports physical therapy* 2008, **38:**542-550.

16. Tiru M, Goh SH, Low BY: **Use of percussion as a screening tool in the diagnosis of occult hip fractures.** *Singapore medical journal* 2002, **43:**467-469.
