# Supplementary material for: A systematic review of the diagnostic performance of orthopedic physical examination tests of the hip
Source: BMC Musculoskelet Disord. 2013 Aug 30;14:257. doi: 10.1186/1471-2474-14-257 (PMC3766647; doi:10.1186/1471-2474-14-257)
Supplement: Additional file 3 — Diagnostic performances of physical test-hip pathology combinations from excluded studies (2×2 contingency tables). File is a table of diagnostic characteristics of physical test-hip pathology combinations (sensitivity, specificity, positive and negative predictive values, and positive and negative likelihood ratios) from excluded studies that allowed for the construction of complete 2×2 contingency tables. [file 1471-2474-14-257-S3.docx]

**Additional file 3. Diagnostic performances of physical test-hip pathology combinations from excluded studies (2x2 contingency tables).**

**Article Title:** A systematic review of the diagnostic performance of orthopedic physical examination tests of the hip.

**Journal**: BMC Musculoskeletal Disorders

**Authors**: Labib A. Rahman^1^; Sam Adie^1,2,3^ ; Justine M. Naylor ^1,2,3^; Rajat Mittal^1,2,3^; Sarah So^1^; Ian A. Harris^1,2,3^

^1^South West Sydney Clinical School, University of New South Wales, ^2^Orthopaedic Department, Liverpool Hospital, ^3^Whitlam Orthopaedic Research Centre

| **Study** | **Test** | | **Pathology** | **Reference Standard** | **Sensitivity** | **Specificity** | **PPV** | **NPV** | **+LR** | **-LR** |  |
| --- | --- | --- | --- | --- | --- | --- | --- | --- | --- | --- | --- |
|  |  |  |  |  | **(95%CI)** | **(95%CI)** |  |  | **(95%CI)** | **(95%CI)** |  |
|  |  |  |  |  | **TP/ (TP+FN)** | **TN/ (TN+FP)** |  |  |  |  |  |
| **Altman et al. 1991 [1]** | Antalgic Gait | | Symptomatic Osteoarthritis | Clinical diagnosis including index test | 0.85 | 0.43 | 0.66 | 0.69 | 1.49 | 0.35 |  |
|  |  |  |  |  | 0.79-0.90 | 0.35-0.50 |  |  | 1.22-1.78 | 0.20-0.59 |  |
|  |  |  |  |  | 79/93 | 31/72 |  |  |  |  |  |
| **Altman et al. 1991 [1]** | Pain on Passive Hip Flexion | | Symptomatic Osteoarthritis | Clinical diagnosis including index test | 0.80 | 0.40 | 0.63 | 0.61 | 1.33 | 0.51 |  |
|  |  |  |  |  | 0.74-0.85 | 0.33-0.47 |  |  | 1.10-1.59 | 0.32-0.79 |  |
| **Altman et al. 1991 [1]** | Pain on Passive Hip Extension | | Symptomatic Osteoarthritis | Clinical diagnosis including index test | 0.64 | 0.50 | 0.63 | 0.51 | 1.28 | 0.72 |  |
|  |  |  |  |  | 0.58-0.71 | 0.41-0.59 |  |  | 0.98-1.70 | 0.50-1.03 |  |
|  |  |  |  |  | 68/106 | 40/80 |  |  |  |  |  |
| **Altman et al. 1991 [1]** | Pain on Passive Hip Abduction | | Symptomatic Osteoarthritis | Clinical diagnosis including index test | 0.76 | 0.44 | 0.64 | 0.59 | 1.36 | 0.55 |  |
|  |  |  |  |  | 0.70-0.81 | 0.37-0.51 |  |  | 1.10-1.67 | 0.36-0.82 |  |
|  |  |  |  |  | 82/108 | 37/84 |  |  |  |  |  |
| **Altman et al. 1991 [1]** | Pain on Passive Hip Adduction | | Symptomatic Osteoarthritis | Clinical diagnosis including index test | 0.68 | 0.54 | 0.66 | 0.57 | 1.49 | 0.59 |  |
|  |  |  |  |  | 0.62-0.74 | 0.46-0.62 |  |  | 1.16-1.93 | 0.42-0.82 |  |
|  |  |  |  |  | 73/107 | 45/83 |  |  |  |  |  |
| **Altman et al. 1991 [1]** | Pain on Passive Hip Internal Rotation | | Symptomatic Osteoarthritis | Clinical diagnosis including index test | 0.82 | 0.39 | 0.64 | 0.63 | 1.36 | 0.45 |  |
|  |  |  |  |  | 0.77-0.87 | 0.32-0.46 |  |  | 1.13-1.61 | 0.28-0.72 |  |
|  |  |  |  |  | 89/108 | 33/84 |  |  |  |  |  |
| **Altman et al. 1991 [1]** | Pain on Passive Hip External Rotation | | Symptomatic Osteoarthritis | Clinical diagnosis including index test | 0.79 | 0.37 | 0.62 | 0.58 | 1.26 | 0.56 |  |
|  |  |  |  |  | 0.74-0.85 | 0.30-0.44 |  |  | 1.05-1.50 | 0.35-0.88 |  |
|  |  |  |  |  | 85/107 | 31/84 |  |  |  |  |  |
| **Altman et al. 1991 [1]** | Trendelenburg Sign | | Symptomatic Osteoarthritis | Clinical diagnosis including index test | 0.37 | 0.81 | 0.73 | 0.47 | 1.90 | 0.78 |  |
|  |  |  |  |  | 0.31-0.42 | 0.72-0.88 |  |  | 1.12-3.34 | 0.67-0.96 |  |
|  |  |  |  |  | 35/95 | 54/67 |  |  |  |  |  |
| **Altman et al. 1991 [1]** | Flexion ROM < 115^o^ | | Symptomatic Osteoarthritis | Radiography | 0.96 | 0.18 | 0.61 | 0.75 | 1.16 | 0.25 | |
|  |  |  |  |  | 0.92-0.98 | 0.13-0.21 |  |  | 1.06-1.24 | 0.10-0.63 | |
|  |  |  |  |  | 109/ 114 | 15/85 |  |  |  |  |  |
| **Altman et al. 1991 [1]** | Internal Rotation ROM < 15^o^ | | Symptomatic Osteoarthritis | Radiography | 0.66 | 0.72 | 0.76 | 0.61 | 2.33 | 0.48 | |
|  |  |  |  |  | 0.60-0.71 | 0.64-0.79 |  |  | 1.67-3.32 | 0.37-0.63 | |
|  |  |  |  |  | 75/114 | 61/85 |  |  |  |  |  |
| **Asayama et al. 2002 [2]** | Trendelenburg Sign | | Osteoarthritis | Radiography (implied) | 1.00 | 1.00 | 1.00 | 1.00 | 35.89 | 0.06 | |
|  |  |  |  |  | 0.80-1.00 | 0.91-1.00 |  |  | 5.73 – 212.06 | 0.01 – 0.31 | |
|  |  |  |  |  | 8/8 | 18/18 |  |  |  |  |  |
| **Brown et al. 2004 [3]** | Pain on Internal Rotation | | Pathology Local to the Hip | Radiography for Hips; and MRI or Radiography for the Spine | 0.58 | 0.78 | 0.92 | 0.30 | 2.63 | 0.53 | |
|  |  |  |  |  | 0.54-0.62 | 0.57-0.91 |  |  | 1.25-6.59 | 0.43-0.81 | |
|  |  |  |  |  | 45/77 | 14/18 |  |  |  |  |  |
| **Brown et al. 2004 [3]** | Antalgic Gait | | Pathology Local to the Hip | Radiography for Hips; and MRI or Radiography for the Spine | 0.39 | 0.83 | 0.91 | 0.24 | 2.34 | 0.73 | |
|  |  |  |  |  | 0.34-0.42 | 0.63-0.94 |  |  | 0.94-6.92 | 0.62-1.04 | |
|  |  |  |  |  | 30/77 | 15/18 |  |  |  |  |  |
| **Brown et al. 2004 [3]** | List | | Pathology Local to the Hip | Radiography for Hips; and MRI or Radiography for the Spine | 0.08 | 0.83 | 0.67 | 0.17 | 0.47 | 1.11 | |
|  |  |  |  |  | 0.04-0.10 | 0.69-0.94 |  |  | 0.14-1.64 | 0.96-1.39 | |
|  |  |  |  |  | 6/77 | 15/18 |  |  |  |  |  |
| **Brown et al. 2004 [3]** | Testing for Fixed Flexion Contraction of the Hip | | Pathology Local to the Hip | Radiography for Hips; and MRI or Radiography for the Spine | 0.29 | 0.94 | 0.96 | 0.24 | 5.14 | 0.76 | |
|  |  |  |  |  | 0.25-0.30 | 0.77-0.99 |  |  | 1.06-29.78 | 0.71-0.98 | |
|  |  |  |  |  | 22/77 | 17/18 |  |  |  |  |  |
| **Cantini et al. 2005 [4]** | FABER Test | | Hip Synovitis | MRI | 0.67 | 1.00 | 1.00 | 0.67 | 22.44 | 0.35 | |
|  |  |  |  |  | 0.57-0.67 | 0.85-1.00 |  |  | 3.01 - 218.51 | 0.32 – 0.55 | |
|  |  |  |  |  | 16/24 | 16/16 |  |  |  |  |  |
| **Cantini et al. 2005 [4]** | Lateral Hip Pain on External Rotation and Abduction | | Inflammation of the Trochanteric Bursa | MRI | 0.97 | 1.00 | 1.00 | 0.67 | 5.77 | 0.05 | |
|  |  |  |  |  | 0.94-0.97 | 0.39-1 |  |  | 1.42 – 52.85 | 0.03 – 0.22 | |
|  |  |  |  |  | 37/38 | 2/2 |  |  |  |  |  |
| **Cantini et al. 2005 [4]** | Pain Aggravated by Extension, Relieved by Flexion | | Inflammation of the Iliopsoas Bursa | MRI | 1.00 | 0.48 | 0.54 | 1.00 | 1.87 | 0.07 | |
|  |  |  |  |  | 0.84-1.00 | 0.38-0.48 |  |  | 1.30 – 1.99 | 0.01 – 0.51 | |
|  |  |  |  |  | 15/15 | 12/25 |  |  |  |  |  |
| **Joe et al. 2002 [5]** | Passive Flexion ROM < 100^o^. Patient Supine. | | Asymptomatic AVN of the Femoral Head | MRI | 0.00 | 0.99 | 0.00 | 0.95 | 1.78 | 0.99 | |
|  |  |  |  |  | 0.00-0.13 | 0.99-0.99 |  |  | 0.17 – 16.68 | 0.85 – 1.02 | |
|  |  |  |  |  | 0/16 | 326/331 |  |  |  |  |  |
| **Joe et al. 2002 [5]** | Passive Extension ROM < 15^o^. Patient Supine. | | Asymptomatic AVN of the Femoral Head | MRI | 0.19 | 0.92 | 0.10 | 0.96 | 2.22 | 0.89 | |
|  |  |  |  |  | 0.07-0.41 | 0.91-0.93 |  |  | 0.74-5.56 | 0.64-1.03 | |
|  |  |  |  |  | 3/16 | 303/331 |  |  |  |  |  |
| **Joe et al. 2002 [5]** | Passive Adduction ROM < 20^o^. Patient Supine. | | Asymptomatic AVN of the Femoral Head | MRI | 0.00 | 0.95 | 0.00 | 0.95 | 0.56 | 1.02 | |
|  |  |  |  |  | 0-0.17 | 0.95-0.96 |  |  | 0.06 – 4.68 | 0.83 – 1.05 | |
|  |  |  |  |  | 0/16 | 314/331 |  |  |  |  |  |
| **Joe et al. 2002 [5]** | Passive Abduction ROM < 45^o^. Patient Supine. | | Asymptomatic AVN of the Femoral Head | MRI | 0.31 | 0.86 | 0.09 | 0.96 | 2.15 | 0.80 | |
|  |  |  |  |  | 0.14-0.55 | 0.85-0.87 |  |  | 0.94-4.07 | 0.53-1.01 | |
|  |  |  |  |  | 5/16 | 283/331 |  |  |  |  |  |
| **Joe et al. 2002 [5]** | Passive Internal Rotation ROM < 15^o^. Patient Supine. | | Asymptomatic AVN of the Femoral Head | MRI | 0.50 | 0.67 | 0.07 | 0.97 | 1.53 | 0.74 | |
|  |  |  |  |  | 0.28-0.72 | 0.66-0.68 |  |  | 0.84-2.27 | 0.42-1.08 | |
|  |  |  |  |  | 8/16 | 223/331 |  |  |  |  |  |
| **Joe et al. 2002 [5]** | Passive External Rotation ROM < 60^o^. Patient Supine. | | Asymptomatic AVN of the Femoral Head | MRI | 0.38 | 0.73 | 0.06 | 0.96 | 1.36 | 0.86 | |
|  |  |  |  |  | 0.19-0.61 | 0.72-0.74 |  |  | 0.66-2.30 | 0.53-1.14 | |
|  |  |  |  |  | 6/16 | 240/331 |  |  |  |  |  |
| **Joe et al. 2002** | Any Abnormal ROM Test in the 6 Planes Described Above. Patient Supine. | | Asymptomatic AVN of the Femoral Head | MRI | 0.69 | 0.46 | 0.06 | 0.97 | 1.28 | 0.68 | |
|  |  |  |  |  | 0.45-0.86 | 0.45-0.47 |  |  | 0.82-1.62 | 0.30-1.22 | |
|  |  |  |  |  | 11/16 | 153/331 |  |  |  |  |  |
| **Joe et al. 2002 [5]** | Pain on Passive Flexion. Patient Supine. | | Asymptomatic AVN of the Femoral Head | MRI | 0.00 | 0.97 | 0.00 | 0.95 | 1.03 | 1.00 | |
|  |  |  |  |  | 0-0.15 | 0.97-0.98 |  |  | 0.10 – 9.09 | 0.83 – 1.03 | |
|  |  |  |  |  | 0/16 | 322/331 |  |  |  |  |  |
| **Joe et al. 2002 [5]** | Pain on Passive Extension. Patient Supine. | | Asymptomatic AVN of the Femoral Head | MRI | 0.00 | 0.99 | 0.00 | 0.95 | 3.91 | 0.98 | |
|  |  |  |  |  | 0-0.08 | 0.99-1 |  |  | 0.35 – 41.89 | 0.88 – 1.01 | |
|  |  |  |  |  | 0/16 | 329/331 |  |  |  |  |  |
| **Joe et al. 2002 [5]** | Pain on Passive Adduction. Patient Supine. | | Asymptomatic AVN of the Femoral Head | MRI | 0.00 | 0.98 | 0.00 | 0.95 | 1.30 | 0.99 | |
|  |  |  |  |  | 0-0.14 | 0.98-0.99 |  |  | 0.13 – 11.82 | 0.84 – 1.02 | |
|  |  |  |  |  | 0/16 | 324/331 |  |  |  |  |  |
| **Joe et al. 2002 [5]** | Pain on Passive Abduction. Patient Supine. | | Asymptomatic AVN of the Femoral Head | MRI | 0.00 | 0.97 | 0.00 | 0.95 | 0.93 | 1.00 | |
|  |  |  |  |  | 0-0.16 | 0.97-0.98 |  |  | 0.09 – 8.14 | 0.83 – 1.03 | |
|  |  |  |  |  | 0/16 | 321/331 |  |  |  |  |  |
| **Joe et al. 2002 [5]** | Pain on Passive Internal Rotation. Patient Supine. | | Asymptomatic AVN of the Femoral Head | MRI | 0.1250 | 0.86 | 0.04 | 0.95 | 0.92 | 1.01 | |
|  |  |  |  |  | 0.04-0.35 | 0.86-0.88 |  |  | 0.25-2.78 | 0.75-1.12 | |
|  |  |  |  |  | 2/16 | 286/331 |  |  |  |  |  |
| **Joe et al. 2002 [5]** | Pain on Passive External Rotation. Patient Supine. | | Asymptomatic AVN of the Femoral Head | MRI | 0.00 | 0.91 | 0.00 | 0.95 | 0.32 | 1.07 | |
|  |  |  |  |  | 0-0.18 | 0.91-0.92 |  |  | 0.03 – 2.60 | 0.86 – 1.10 | |
|  |  |  |  |  | 0/16 | 301/331 |  |  |  |  |  |
| **Joe et al. 2002 [5]** | Pain on Any Passive Motion Test in the 6 Planes Described Above. Patient Supine. | | Asymptomatic AVN of the Femoral Head | MRI | 0.13 | 0.71 | 0.02 | 0.94 | 0.43 | 1.24 | |
|  |  |  |  |  | 0.04-0.35 | 0.70-0.72 |  |  | 0.12-1.25 | 0.90-1.37 | |
|  |  |  |  |  | 2/16 | 234/331 |  |  |  |  |  |
| **Joe et al. 2002 [5]** | Pain Complex^a^ | | Asymptomatic AVN of the Femoral Head | MRI | 0.25 | 0.71 | 0.04 | 0.95 | 0.86 | 1.06 | |
|  |  |  |  |  | 0.10-0.49 | 0.70-0.72 |  |  | 0.35-1.75 | 0.71-1.28 | |
|  |  |  |  |  | 4/16 | 235/331 |  |  |  |  |  |
| **Joe et al. 2002 [5]** | Exam Complex ^b^ | | Asymptomatic AVN of the Femoral Head | MRI | 0.88 | 0.34 | 0.06 | 0.98 | 1.33 | 0.37 | |
|  |  |  |  |  | 0.65-0.97 | 0.33-0.35 |  |  | 0.97-1.48 | 0.10-1.07 | |
|  |  |  |  |  | 14/16 | 113/331 |  |  |  |  |  |
| **Klässbo et al. 2003 [6]** | Passive Flexion ROM <110^o^ (Mean for symptom-free hips) | | Symptomatic Osteoarthritis | Radiography | 0.26 | 0.92 | 0.73 | 0.58 | 3.04 | 0.81 | |
|  |  |  |  |  | 0.22-0.29 | 0.88-0.95 |  |  | 1.78-5.28 | 0.75-0.89 | |
|  |  |  |  |  | 41/159 | 162/177 |  |  |  |  |  |
| **Klässbo et al. 2003 [6]** | Passive Internal Rotation ROM < 20^o^ (Mean for symptom-free hips) | | Symptomatic Osteoarthritis | Radiography | 0.12 | 0.95 | 0.68 | 0.55 | 2.35 | 0.93 | |
|  |  |  |  |  | 0.09-0.14 | 0.92-0.97 |  |  | 1.12-5.00 | 0.88-0.99 | |
|  |  |  |  |  | 19/159 | 168/177 |  |  |  |  |  |
| **Klässbo et al. 2003 [6]** | Passive Internal Rotation ROM < 20^o^ and Flexion ROM <110^o^ (Mean for symptom-free hips) | | Symptomatic Osteoarthritis | Radiography | 0.03 | 0.99 | 0.83 | 0.53 | 5.57 | 0.97 | |
|  |  |  |  |  | 0.02-0.04 | 0.98-1.00 |  |  | 0.87-35.87 | 0.96-1.00 | |
|  |  |  |  |  | 5/159 | 176/177 |  |  |  |  |  |
| **Klässbo et al. 2003 [6]** | Passive Abduction ROM <20^o^ (Mean for symptom-free hips) | | Symptomatic Osteoarthritis | Radiography | 0.01 | 0.93 | 0.14 | 0.51 | 0.19 | 1.06 | |
|  |  |  |  |  | 0.00-0.04 | 0.92-0.95 |  |  | 0.05-0.72 | 1.01-1.08 | |
|  |  |  |  |  | 2/159 | 165/177 |  |  |  |  |  |
| **Klässbo et al. 2003 [6]** | | Passive Abduction ROM <20^o^, Flexion ROM <110^o^ and Internal Rotation ROM < 20^o^  (Mean for symptom-free hips) | Symptomatic Osteoarthritis | Radiography | 0.07 | 0.99 | 0.92 | 0.54 | 12.25 | 0.94 | |
|  |  |  |  |  | 0.05-0.07 | 0.98-1.00 |  |  | 2.08-73.66 | 0.93-0.97 | |
|  |  |  |  |  | 11/159 | 176/177 |  |  |  |  |  |
| **Klässbo et al. 2003 [6]** | Limited Passive ROM in All 6 Planes (Mean for symptom-free hips) | | Symptomatic Osteoarthritis | Radiography | 0.04 | 1.00 | 1.00 | 0.54 | 14.46 | 0.96 | |
|  |  |  |  |  | 0.02-0.04 | 0.99-1.00 |  |  | 1.45 – 147.09 | 0.96 – 0.99 | |
|  |  |  |  |  | 6/ 159 | 177/ 177 |  |  |  |  |  |
| **Lequesne et al. 2008 [7]** | | Pain on Single-Leg Stance Within 30 Seconds | Anterior Gluteus Medius Tendon Tear | MRI | 1.00 | 0.95 | 0.89 | 1.00 | 15.53 | 0.03 | |
|  |  |  |  |  | 0.86-1.00 | 0.89-0.95 |  |  | 6.68 – 19.42 | 0.00 – 0.20 | |
|  |  |  |  |  | 16/16 | 37/39 |  |  |  |  |  |
| **Lequesne et al. 2008 [7]** | | Pain on Single-Leg Stance Within 30 Seconds | Gluteus Minimus Tendon Tear | MRI | 1.00 | 0.71 | 0.17 | 1.00 | 2.99 | 0.18 | |
|  |  |  |  |  | 0.45-1.00 | 0.68-0.71 |  |  | 1.26 -3.47 | 0.02 -0.88 | |
|  |  |  |  |  | 3/3 | 37/52 |  |  |  |  |  |
| **Lequesne et al. 2008 [7]** | | Pain on Single-Leg Stance Within 30 Seconds | Tendinitis of the Anterior Gluteus Medius and/or Gluteus Minimus Tendons | MRI | 1.00 | 0.93 | 0.83 | 1.00 | 11.35 | 0.03 | |
|  |  |  |  |  | 0.85-1.00 | 0.87-0.93 |  |  | 5.52 – 13.38 | 0.00 – 0.22 | |
|  |  |  |  |  | 15/15 | 37/40 |  |  |  |  |  |
| **Lequesne et al. 2008 [7]** | | Pain on Single-Leg Stance Within 30 Seconds | Bursitis of the Trochanteric, Sub-Gluteus Medius and/or sub-Gluteus Minimus Bursae | MRI | 1.00 | 0.95 | 0.89 | 1.00 | 15.53 | 0.03 | |
|  |  |  |  |  | 0.89-0.95 | 0.77-0.89 |  |  | 6.68 – 19.42 | 0.00 – 0.20 | |
|  |  |  |  |  | 16/16 | 37/39 |  |  |  |  |  |
| **Leunig et al. 2004 [8]** | Impingement Test | | Acetabular Labral Tears | MRA | 1.00 | 0.00 | 0.64 | - | 1.02 | 0.58 | |
|  |  |  |  |  | 1.00-1.00 | 0.00-0.00 |  |  | 0.96 – 1.09 | 0.03 – 9.95 | |
|  |  |  |  |  | 18/18 | 0/10 |  |  |  |  |  |
| **Leunig et al. 2004 [8]** | Impingement Test | | Acetabular Labral Hypertrophy | MRA | 1.00 | 0.00 | 0.43 | - | 0.99 | 1.31 | |
|  |  |  |  |  | 1.00-1.00 | 0.00-0.00 |  |  | 0.93 – 1.05 | 0.07 – 22.49 | |
|  |  |  |  |  | 12/12 | 0/16 |  |  |  |  |  |
| **Leunig et al. 2004 [8]** | Impingement Test | | Presence of Soft Tissue Ganglia in the Acetabular Labrum | MRA | 1.00 | 0.00 | 0.46 | - | 1.00 | 1.14 | |
|  |  |  |  |  | 1.00-1.00 | 0.00-0.00 |  |  | 0.94 – 1.06 | 0.07 – 19.65 | |
|  |  |  |  |  | 13/13 | 0/15 |  |  |  |  |  |
| **Lohan et al. 2009 [9]** | Impingement Test | | Cam-Type Femoroacetabular Impingement | Surgery | 0.77 | 0.87 | 0.86 | 0.79 | 6.00 | 0.26 |  |
|  |  |  |  |  | 0.67-0.83 | 0.77-0.94 |  |  | 2.97-13.02 | 0.18-0.43 |  |
|  |  |  |  |  | 30/39 | 34/39 |  |  |  |  |  |
| **Martin et al. 2008 [10]** | FABER Test | | Intra-articular Hip Pathology | Diagnostic / Therapeutic Intra-articular Hip Injection | 0.60 | 0.18 | 0.45 | 0.29 | 0.73 | 2.20 | |
|  |  |  |  |  | 0.51-0.72 | 0.08-0.32 |  |  | 0.56-1.07 | 0.86-6.07 | |
|  |  |  |  |  | 15/25 | 4/22 |  |  |  |  |  |
| **Martin et al. 2008 [10]** | Impingement Test | | Intra-articular Hip Pathology | Diagnostic / Therapeutic Intra-articular Hip Injection | 0.78 | 0.10 | 0.53 | 0.25 | 0.86 | 2.33 | |
|  |  |  |  |  | 0.73-0.87 | 0.03-0.22 |  |  | 0.75-1.11 | 0.60-9.78 | |
| **Maslowski et al. 2010 [11]** | FABER Test | | Intra-articular Hip Pathology | Diagnostic / Therapeutic Intra-articular Hip Injection (Visual Analog Scale) | 0.59 | 0.32 | 0.41 | 0.50 | 0.87 | 1.27 | |
|  |  |  |  |  | 0.45-0.74 | 0.21-0.44 |  |  | 0.57-1.30 | 0.61-2.61 | |
|  |  |  |  |  | 13/22 | 9/28 |  |  |  |  |  |
| **Maslowski et al. 2010 [11]** | Impingement Test (Internal Rotation Over Pressure) | | Intra-articular Hip Pathology | Diagnostic / Therapeutic Intra-articular Hip Injection (Visual Analog Scale) | 0.91 | 0.18 | 0.47 | 0.71 | 1.11 | 0.51 | |
|  |  |  |  |  | 0.80-0.97 | 0.10-0.23 |  |  | 0.89-1.26 | 0.12-2.08 | |
|  |  |  |  |  | 20/22 | 5/28 |  |  |  |  |  |
| **Maslowski et al. 2010 [11]** | Stinchfield Maneuvre | | Intra-articular Hip Pathology | Diagnostic / Therapeutic Intra-articular Hip Injection (Visual Analog Scale) | 0.68 | 0.32 | 0.41 | 0.50 | 0.87 | 1.27 | |
|  |  |  |  |  | 0.54-0.82 | 0.21-0.43 |  |  | 0.69-1.42 | 0.43-2.18 | |
|  |  |  |  |  | 15/22 | 9/28 |  |  |  |  |  |
| **Pritchard et al. 2012 [12]** | Ligamentum teres (LT) test | | Ligamentum teres pathology (tears or synovitis) | Arthroscopy | 0.88 | 0.69 | 0.78 | 0.82 | 2.84 | 0.18 | |
|  |  |  |  |  | 0.69 – 0.98 | 0.46 – 0.82 |  |  | 1.27 – 5.30 | 0.03 – 0.69 | |
|  |  |  |  |  | 14/16 | 9/13 |  |  |  |  |  |
| **Robb et al. 2009 [13]** | Impingement Test | | Acetabular Retroversion | Radiography | 0.00 | 0.79 | 0.00 | 0.85 | 0.71 | 1.09 | |
|  |  |  |  |  | 0.00-0.60 | 0.79-0.87 |  |  | 0.07 – 4.49 | 0.43 – 1.33 | |
|  |  |  |  |  | 0/2 | 11/14 |  |  |  |  |  |
| **Troelsen et al. 2009 [14]** | Impingement Test | | Acetabular Labral Tears | MRA | 0.59 | 1.00 | 1.00 | 0.13 | 2.33 | 0.56 | |
|  |  |  |  |  | 0.54-0.59 | 0.21-1.00 |  |  | 0.66 – 22.57 | 0.40 – 2.30 | |
|  |  |  |  |  | 10/17 | 1/1 |  |  |  |  |  |
| **Troelsen et al. 2009 [14]** | FABER Test | | Acetabular Labral Tears | MRA | 0.41 | 1.00 | 1.00 | 0.09 | 1.67 | 0.78 | |
|  |  |  |  |  | 0.37-0.41 | 0.21-1.00 |  |  | 0.45 – 16.39 | 0.57 – 3.09 | |
|  |  |  |  |  | 7/17 | 1/1 |  |  |  |  |  |
| **Troelsen et al. 2009 [14]** | Resisted Straight Leg Raise | | Acetabular Labral Tears | MRA | 0.06 | 1.00 | 1.00 | 0.06 | 0.33 | 1.22 | |
|  |  |  |  |  | 0.02-0.06 | 0.31-1.00 |  |  | 0.04 – 3.95 | 0.92 – 3.47 | |
| **Woodley et al. 2008 [15]** | | Pain on Active Hip Internal Rotation | Pathology of the Gluteus Medius or Gluteus Minimus Tendons | MRI | 0.33 | 0.86 | 0.83 | 0.38 | 2.33 | 0.78 | |
|  |  |  |  |  | 0.20-0.39 | 0.58-0.97 |  |  | 0.48-14.58 | 0.63-1.38 | |
|  |  |  |  |  | 5/15 | 6/7 |  |  |  |  |  |
| **Woodley et al. 2008 [15]** | | Pain on Passive Hip Abduction | Pathology of the Gluteus Medius or Gluteus Minimus Tendons | MRI | 0.60 | 1.00 | 1.00 | 0.54 | 9.50 | 0.43 | |
|  |  |  |  |  | 0.47-0.60 | 0.72-1.00 |  |  | 1.37 – 93.70 | 0.38 – 0.81 | |
|  |  |  |  |  | 9/15 | 7/7 |  |  |  |  |  |
| **Woodley et al. 2008 [15]** | | Pain on Passive Hip Internal Rotation | Pathology of the Gluteus Medius or Gluteus Minimus Tendons | MRI | 0.53 | 0.86 | 0.89 | 0.46 | 3.73 | 0.54 | |
|  |  |  |  |  | 0.39-0.59 | 0.56-0.97 |  |  | 0.88-22.11 | 0.42-1.10 | |
|  |  |  |  |  | 8/15 | 6/7 |  |  |  |  |  |
| **Woodley et al. 2008 [15]** | | Pain on Resisted Testing of the Gluteus Minimus Muscle | Pathology of the Gluteus Medius or Gluteus Minimus Tendons | MRI | 0.47 | 0.86 | 0.88 | 0.43 | 3.27 | 0.62 | |
|  |  |  |  |  | 0.33-0.52 | 0.56-0.97 |  |  | 0.74-19.6 | 0.49-1.20 | |
|  |  |  |  |  | 7/15 | 6/7 |  |  |  |  |  |
| **Woodley et al. 2008 [15]** | | Pain on Resisted Tests of Both the Gluteus Medius and Gluteus Minimus Muscle | Pathology of the Gluteus Medius or Gluteus Minimus Tendons | MRI | 0.47 | 0.86 | 0.88 | 0.43 | 3.27 | 0.62 | |
|  |  |  |  |  | 0.33-0.52 | 0.56-0.97 |  |  | 0.74-19.6 | 0.49-1.20 | |
|  |  |  |  |  | 7/15 | 6/7 |  |  |  |  |  |
| **Woodley et al. 2008 [15]** | | Trendelenburg Sign | Pathology of the Gluteus Medius or Gluteus Minimus Tendons | MRI | 0.20 | 1.00 | 1.00 | 0.37 | 3.50 | 0.83 | |
|  |  |  |  |  | 0.10-0.20 | 0.78-1.00 |  |  | 0.39 – 37.07 | 0.76 – 1.24 | |
|  |  |  |  |  | 3/15 | 7/7 |  |  |  |  |  |
| **Youdas et al. 2010 [16]** | Trendelenburg Sign (Adduction of Pelvis-on-Femur Angle) | | Osteoarthritis | Radiography | 0.55 | 0.70 | 0.65 | 0.61 | 1.83 | 0.64 | |
|  |  |  |  |  | 0.40-0.68 | 0.55-0.83 |  |  | 0.88-3.96 | 0.39-1.10 | |
|  |  |  |  |  | 11/20 | 14/20 |  |  |  |  |  |
| **Youdas et al. 2010 [16]** | Isometric Manual Muscle Test < 30% body weight | | Osteoarthritis | Radiography | 0.35 | 0.90 | 0.78 | 0.58 | 3.50 | 0.72 | |
|  |  |  |  |  | 0.22-0.42 | 0.77-0.97 |  |  | 0.96-14.17 | 0.60-1.01 | |
|  |  |  |  |  | 7/20 | 18/20 |  |  |  |  |  |
| **Zeren et al. 2006 [17]** | Active Range of Motion Test (Pain on Active Hip Extension with an Extended Knee; Active Pain on Knee Flexion) | | Biceps Femoris Muscle-Strain Injuries | Ultrasonography | 0.55 | 1.00 | 1.00 | 0.69 | 155.00 | 0.45 | |
|  |  |  |  |  | 0.53-0.55 | 0.98-1.00 |  |  | 17.25 – 1490.98 | 0.45 – 0.49 | |
|  |  |  |  |  | 77/ 140 | 140/ 140 |  |  |  |  |  |
| **Zeren et al. 2006 [17]** | Passive Range of Motion Test (Pain on Passive Hip Flexion; Pain on Passive Knee Extension) | | Biceps Femoris Muscle-Strain Injuries | Ultrasonography | 0.57 | 1.00 | 1.00 | 0.70 | 161.00 | 0.43 | |
|  |  |  |  |  | 0.55-0.57 | 0.98-1.00 |  |  | 17.96 – 1548.37 | 0.43 – 0.47 | |
|  |  |  |  |  | 80/ 140 | 140/ 140 |  |  |  |  |  |
| **Zeren et al. 2006 [17]** | Resisted Range of Motion Tests (Pain on Resisted Hip Extension with an Extended Knee, Pain on Resisted Hip Rotation in the Neural Position; Pain on Knee Flexion) | | Biceps Femoris Muscle-Strain Injuries | Ultrasonography | 0.61 | 1.00 | 1.00 | 0.72 | 171.00 | 0.40 | |
|  |  |  |  |  | 0.58-0.61 | 0.98-1.00 |  |  | 19.16 – 1643.85 | 0.39 – 0.43 | |
|  |  |  |  |  | 85/ 140 | 140/ 140 |  |  |  |  |  |
| **Zeren et al. 2006 [17]** | Taking Off the Shoe Test | | Biceps Femoris Muscle-Strain Injuries | Ultrasonography | 1.00 | 1.00 | 1.00 | 1.00 | 281.00 | 0.00 | |
|  |  |  |  |  | 0.99-1.00 | 0.99-1.00 |  |  | 49.48 – 1595.80 | 0.00 – 0.02 | |
|  |  |  |  |  | 140/ 140 | 140/ 140 |  |  |  |  |  |

Positive Predictive Value (PPV), Negative Predictive Value (NPV), Positive Likelihood Ratio (+LR), Negative Likelihood Ratio (-LR), 95% Confidence Interval (95%CI), True Positives (TP), False Positives (FP), True Negatives (TN), False Negatives (FN), Range of Motion (ROM). All values rounded to 2 decimal places. When one of the cells of the 2x2 contingency table contained the value ‘zero’, we added 0.5 to each cell in order to calculate likelihood ratio values and their confidence intervals.

^a^ Pain complex was defined as: pain on any passive motion test in 6 planes; or pain on provocative tests including Patrick's test, Thomas test, Ober's test, straight leg raise, axial loading maneuver, femoral head compression test and distraction in the supine position with leg extended; or single leg stand for 2 minutes or single leg hip for 10-20 repetitions

^b^ Exam complex was defined as: restricted passive range of motion in any of 6 planes (flexion < 100^o^, extension < 15^o^, adduction < 20^o^, abduction < 45^o^, internal rotation <15^o^ or external rotation < 60^o^) or pain complex, which was defined as: pain on any passive motion test in 6 planes; or pain on provocative tests including Patrick's test, Thomas test, Ober's test, straight leg raise, axial loading maneuver, femoral head compression test and distraction in the supine position with leg extended; or single leg stand for 2 minutes or single leg hip for 10-20 repetitions

**References**:

1. Altman R, Alarcon G, Appelrouth D, Bloch D, Borenstein D, Brandt K, Brown C, Cooke TD, Daniel W, Feldman D, et al.: **The American College of Rheumatology criteria for the classification and reporting of osteoarthritis of the hip.** *Arthritis and rheumatism* 1991, **34:**505-514.

2. Asayama I, Naito M, Fujisawa M, Kambe T: **Relationship between radiographic measurements of reconstructed hip joint position and the Trendelenburg sign.** *The Journal of arthroplasty* 2002, **17:**747-751.

3. Brown MD, Gomez-Marin O, Brookfield KF, Li PS: **Differential diagnosis of hip disease versus spine disease.** *Clinical orthopaedics and related research* 2004**:**280-284.

4. Cantini F, Niccoli L, Nannini C, Padula A, Olivieri I, Boiardi L, Salvarani C: **Inflammatory changes of hip synovial structures in polymyalgia rheumatica.** *Clinical and experimental rheumatology* 2005, **23:**462-468.

5. Joe GO, Kovacs JA, Miller KD, Kelly GG, Koziol DE, Jones EC, Mican JM, Masur H, Gerber L: **Diagnosis of avascular necrosis of the hip in asymptomatic HIV-infected patients: Clinical correlation of physical examination with magnetic resonance imaging.** *Journal of back and musculoskeletal rehabilitation* 2002, **16:**135-139.

6. Klassbo M, Harms-Ringdahl K, Larsson G: **Examination of passive ROM and capsular patterns in the hip.** *Physiotherapy research international : the journal for researchers and clinicians in physical therapy* 2003, **8:**1-12.

7. Lequesne M, Mathieu P, Vuillemin-Bodaghi V, Bard H, Djian P: **Gluteal tendinopathy in refractory greater trochanter pain syndrome: diagnostic value of two clinical tests.** *Arthritis and rheumatism* 2008, **59:**241-246.

8. Leunig M, Werlen S, Ungersbock A, Ito K, Ganz R: **Evaluation of the acetabular labrum by MR arthrography.** *The Journal of bone and joint surgery British volume* 1997, **79:**230-234.

9. Lohan DG, Seeger LL, Motamedi K, Hame S, Sayre J: **Cam-type femoral-acetabular impingement: is the alpha angle the best MR arthrography has to offer?** *Skeletal radiology* 2009, **38:**855-862.

10. Martin RL, Irrgang JJ, Sekiya JK: **The diagnostic accuracy of a clinical examination in determining intra-articular hip pain for potential hip arthroscopy candidates.** *Arthroscopy : the journal of arthroscopic & related surgery : official publication of the Arthroscopy Association of North America and the International Arthroscopy Association* 2008, **24:**1013-1018.

11. Maslowski E, Sullivan W, Forster Harwood J, Gonzalez P, Kaufman M, Vidal A, Akuthota V: **The diagnostic validity of hip provocation maneuvers to detect intra-articular hip pathology.** *PM & R : the journal of injury, function, and rehabilitation* 2010, **2:**174-181.

12. Pritchard MG, O'Donnell J M, Singh PJ, Bates D: **Clinical examination of the ligamentum teres - A description and validation of the LT test.** *Arthroscopy - Journal of Arthroscopic and Related Surgery* 2012, **2):**e66-e67.

13. Robb CA, Datta A, Nayeemuddin M, Bache CE: **Assessment of acetabular retroversion following long term review of Salter's osteotomy.** *Hip international : the journal of clinical and experimental research on hip pathology and therapy* 2009, **19:**8-12.

14. Troelsen A, Mechlenburg I, Gelineck J, Bolvig L, Jacobsen S, Soballe K: **What is the role of clinical tests and ultrasound in acetabular labral tear diagnostics?** *Acta orthopaedica* 2009, **80:**314-318.

15. Woodley SJ, Nicholson HD, Livingstone V, Doyle TC, Meikle GR, Macintosh JE, Mercer SR: **Lateral hip pain: findings from magnetic resonance imaging and clinical examination.** *The Journal of orthopaedic and sports physical therapy* 2008, **38:**313-328.

16. Youdas JW, Madson TJ, Hollman JH: **Usefulness of the Trendelenburg test for identification of patients with hip joint osteoarthritis.** *Physiotherapy theory and practice* 2010, **26:**184-194.

17. Zeren B, Oztekin HH: **A new self-diagnostic test for biceps femoris muscle strains.** *Clinical journal of sport medicine : official journal of the Canadian Academy of Sport Medicine* 2006, **16:**166-169.
