# Supplementary material for: A systematic review of the diagnostic performance of orthopedic physical examination tests of the hip
Source: BMC Musculoskelet Disord. 2013 Aug 30;14:257. doi: 10.1186/1471-2474-14-257 (PMC3766647; doi:10.1186/1471-2474-14-257)
Supplement: Additional file 4 — Overview of excluded studies and case reports not presented in complete 2×2 contingency tables. File is a basic description of studies and case reports that were excluded from our studies and did not allow for the construction of complete 2×2 contingency tables (for example, because they excluded patients with negative index tests from their study). [file 1471-2474-14-257-S4.docx]

**Additional file 4. Overview of excluded studies and case reports not presented in complete 2x2 contingency tables.**

| No. | Article | Hip Pathology | Index Test(s) | Reference Test(s) |
| --- | --- | --- | --- | --- |
|  | Adkins et al. (2001) | Insufficiency fracture of the acetabular roof in Paget’s disease | Antalgic gait; Stinchfield test; FABER test; pain on internal rotation | Radiography, MRI, CT |
|  | Amstutz et al. (1984) | Greater trochanteric nonunion after total hip arthroplasty | Trendelenburg sign | Radiography |
|  | Amstutz et al. (1978) | Greater trochanteric non-union | Trendelenburg sign | Radiography |
|  | Atilla et al. (2008) | Acetabular fracture after total hip arthroplasty | Leg length (Shortening) | Radiography |
|  | Austin et al. (2008) | Labral tear | Scour test; log roll test; impingement test; FABER test | MRA |
|  | Beaulé et al. (2005) | Femoroacetabular impingement; labral tears | Antalgic gait; leg length discrepancy; impingement test | Radiography; MRA |
|  | Beck et al. (2004) | Femoroacetabular impingement and labral tears | Impingement test | MRA; arthroscopy |
|  | Bewyer et al. (2005) | Tear at musculotendinous junction of gluteus medius muscle | Trendelenburg sign | MRI |
|  | Bhinda et al. (2003) | Dislocation of total hip arthroplasty | Limb shortening | Radiography |
|  | Binningsley (2003) | Acetabular labral tear | Pain on resisted internal rotation; pain on resisted flexion | MRA; Arthroscopy |
|  | Bizzini et al. (2007) | Cam-type femoroacetabular impingement with associated labral lesions | Impingement test | MRA |
|  | Bohannon Mason (2001) | Anterior radial flap of the acetabular labrum | External rotation Thomas test; Internal rotation Thomas test | Arthroscopy |
|  |  | Developmental dysplagia of the hip; chrondromalacia of the weigh-bearing surface of the femoral head; tear of the inferior aspect of the acetabular labrum | Pain and distinct catching sensation with adduction and internal rotation; Thomas test in external rotation; Thomas internal rotation test | Arthroscopy; radiography |
|  | Botti et al. (2005) | Femoral component fracture after THA | Shortened limb | Radiography; confirmation at surgery |
|  | Braun-Moscovici et al. (2006) | Calcific tendinitis of the rectus femoris | Ely’s test | Radiography; bone scintigraphy |
|  | Browder et al. (2005) | Large soft tissue tumour (non-Hodgkinson’s lymphoma) | Trendelenburg limp; pain on flexion; pain on external rotation; empty end feel | Radiography, MRA |
|  | Brunner et al. (2009) | Cam-type femoroacetabular impingement | Impingement Test | MRI |
|  | Burnett et al. (2006) | Acetabular labral tears | Trendelenburg sign; impingement test | Radiography; MRA |
|  | Byrd (1996) | Labral lesions | Painful pop on flexion combined with rotational motion; anterior and lateral hip pain with extreme passive external rotation; painful click with active rotational motion | Arthroscopy |
|  | Chan et al. (2005) | Acetabular labral tear | Pain elicited with patient supine, hip flexed at 90 degrees on internal rotation | Hip arthroscopy |
|  | Chen et al. (2006) | Contracture of the gluteus maximus muscle | Active squat test; hip flexion test | MRI |
|  | Cibere et al. (2008) | Hip osteoarthritis | Trendelenburg sign; true leg length discrepancy; apparent leg length discrepancy; pain on flexion, external rotation and internal rotation; log roll test, Patrick’s test | Radiography |
|  | Clohisy et al. (2009) | Femoroacetabular impingement | FABER/Patrick’s test; resisted straight leg raise; log roll test; anterior impingement test; posterior impingement test | Radiography |
|  | Crevenna et al. (2002) | Calcific bursitis trochanterica | Patrick-Fabere test; pain on flexion; pain on extension; pain on abduction; pain on adduction; pain on external rotation | Radiography |
|  | Diez (2004) | Bilateral congenital hip dislocation | Trendelenburg sign | Radiography |
|  | Eijer et al. (2011) | Acetabular labral lesions and cartilage damage | FADIR test (Anterior Impingement Test) | MRI, Intraoperative confirmation |
|  | Eijer et al. (2001) | Anterior femoroacetabular impingement | Antalgic gait; anterior impingement test; pain on abduction of the extended hip; apparent leg length | Radiography, MRA |
|  | Erhard et al. (2004) | Destructive lesion (metastatic tumour from primary pulmonary adenocarcinoma) of the acetabulum, posterior column, and ischium with soft tissue involvement of the obturator internus muscle | Patrick’s test; passive straight leg-raise | MRI, CT |
|  | File et al. (1988) | Hip fracture | Auscultatory percussion technique | Radiography; CT |
|  | Fisher et al. (2007) | Rupture of the gluteus minimus muscle and at least a partial tear of the gluteus medius | Antalgic gait; limb-length discrepancy; Trendelenburg sign; straight leg-raise | MRI |
|  | Fitzgerald (1995) | Acetabular labral tear | Anterior and posterior Fitzgerald tests | Arthrogram; intra-articular injection; MRI |
|  | Fonstad et al. (2008) | Acetabular labral tears | Impingement test | Intra-articular hip block; MRA |
|  | Fraitzl et al. (2007) | Femoroacetabular impingement in patients previously treated for unilateral slipped capital femoral epiphysis | Anterior impingement test | Radiography |
|  | Gedouin et al. (2010) | Femoroacetabular impingement | Impingement test | Radiography |
|  | Gille et al. (2004) | Primary synovial chondromatosis of the hip | Pain on internal rotation; pain on external rotation | Ultrasonography; radiography; MRI; histology; immunochemistry |
|  | Guanche et al. (2005) | Acetabular labral tear | Limb length discrepancy; pain and reproduction of symptoms with forced flexion with internal rotation of the hip | Arthroscopy |
|  | Guis-Sabatier et al. (1999) | Obturator muscle abscess | Pain on flexion; pain on extension | MRI, CT |
|  | Gurney et al. (2006) | Nondisplaced femoral neck and head stress fracture | Antalgic gait; Trendelenburg sign; FABER test; painful resisted flexion; painful resisted adduction; painful resisted abduction | MRI; bone scan |
|  | Hartmann et al. (2009) | Cam-type femoroacetabular impingement and mild pincer impingement; cam impingement alone with isolated anterolateral offset reduction | Anterior impingement test | Radiography; MRI |
|  | Hase et al. (1999) | Acetabular labral tear | Pain elicited by internal rotation of the hip joint flexed in 90 degrees | Arthroscopy |
|  | Ichikawa et al. (2008) | Fatigue fracture of the bilateral femoral neck | Pain on internal rotation; pain on external rotation | Radiography; MRI |
|  | Ito et al. (2004) | Femoroacetabular impingement (cam-type, pincer or mixed injury mechanism); degenerated or ruptured labra or both | Impingement test | Radiography; MRA; surgical observations |
|  | Ito et al. (2001) | Abnormality of the labrum | Impingement test | MRA |
|  | James et al. (2007) | Femoroacetabular impingement | Impingement Test | CT; MRI |
|  | Kagan (1991) | Lamellar tearing and tedinous degeneration of the gluteus medius tendon at its insertion | Trendelenburg sign | MRI; Surgery |
|  | Kandemir et al. (2003) | Calcific tendinitis of the gluteus medius and minimus | Antalgic gait, Trendelenburg sign | Radiography; fluoroscopic imaging |
|  | Keeney et al. (2004) | Acetabular labral tears | Impingment test | Radiography, CT |
|  | King et al. (2003) | Primary obturator pyomyositis | Pain on active straight leg-raise | MRI; surgical exploration |
|  | Klässbo et al. (2003) | Passive Abduction ROM <20^o^, Flexion ROM <110^o^, Internal Rotation Internal Rotation ROM < 20^o^ and Extension ROM < 5^o^ | Osteoarthritis | Radiography |
|  |  | Passive Abduction ROM <20^o^, Flexion ROM <110^o^, Internal Rotation Internal Rotation ROM < 20^o^, Extension ROM < 5^o^ and External Rotation ROM <25^o^ | Osteoarthritis | Radiography |
|  | LaBan et al. (2004) | Full thickness tear of the gluteus medius tendon with scar formation at its tendinous insertion; effusion of the hip | Straight leg-raise, Patrick’s sign; Duchenne’s antalgic gait | MRI |
|  | Larson et al. (2008) | Femoroacetabular impingement | Impingement test | Radiography, MRA, CT |
|  | Lasanianos et al. (2009) | Occult acetabular fracture complicated by an ipsilateral femoral neck fracture occurring within two months | Shortened limb | Radiography, CT |
|  | Lavigne et al. (2008) | Post-operative hip impingement after hip resurfacing | Antalgic limp; impingement test | Radiography |
|  | Lequesne et al. (2008) | Gluteus medius tendon tear (main posterior tendon and lateral part); gluteus medius tendon tear; bursitis (trochanteric bursitis; subgluteus medius bursitis; subgluteus minimus bursitis; all three) | Pain in external rotation of thigh flexed 90 degrees; single leg stance; resisted external derotation; pain on resisted abduction | MRI; surgery |
|  | Liu et al. (1999) | Iliac muscle abscess | Psoas sign; Patrick test; pain on passive extension; pain on active flexion | Ultrasonography; MRI |
|  | Londers el al. (2007) | Intra-articular hip pathology (mainly labral tears, cartilage lesions) | FADIR test (flexion, adduction and internal rotation test) | Hip arthroscopy |
|  | Malas et al. (2007) | Partial tear of the anterior labrum with perilabral cyst | Pain on external rotation | MRI |
|  | Matsuda (2009) | Severe labrochondral damage | Anterior impingement sign | Hip arthroscopy |
|  | McCarthy et al. (2003) | Avascular necrosis of the femoral head | Hip extension test; impingement test; resisted straight leg-raise; Trendelenburg sign; leg length; log roll test | Radiography; MRI; Arthroscopy |
|  | McCarthy et al. (1995) | Chondral defect of the acetabulum adjacent to a torn, frayed acetabular labrum | Thomas test | Arthroscopy |
|  | McMichael et al. (2009) | Intertrochanteric fracture | Leg length | Radiography |
|  | Miozzari et al. (2010) | Abductor avulsion after total hip arthroplasty | Trendeleburg sign | MRI; ultrasonography; confirmed at surgery |
|  | Mitchell et al. (2003) | Hip joint pathology | Painful hip quadrant compared to the contralateral hip; FABER test | Radiography, ultrasonography; MRA; arthroscopy |
|  | Notzli et al. (2002) | Degenerative labrum/labral tear | Impingement test | Radiography; MRA |
|  | Nunley et al. (2011) | Symptomatic acetabular dysplasia | Presence of limp when walking a distance, Trendelenburg sign, impingement test | Radiography, periacetabular osteotomy |
|  | Padhy et al. (2009) | Primary synovial chondromatosis with femoroacetabular impingement and labral tear | Impingement testl flexion abduction external rotation test; leg length | Radiography; MRI; CT |
|  | Philippon et al. (2007) | Femoroacetabular impingement | Anterior impingement test; FABER test | Radiography |
|  | Pozzi et al. (2009) | Femoroacetabular impingement | Impingement Test | Radiography; MRI |
|  | Quarrier et al. (1998) | Lesser trochanter bony avulsion with ossicle entirely loose and embedded in the iliopsoas tendon near its insertion in the lesser trochanter | Painful passive hip flexion; painful passive hip abduction painful passive external rotation; painful active adduction; painful active internal rotation; painful active external rotation painful active flexion; painful resisted hip flexion; developpe; Thomas test; Ober test; supine to sit test; Patrick’s test; scouring test | Surgical exploration |
|  | Quintos-Macasa et al. (2006) | Transient synovitis of the hip | Painful flexion, extension, abduction, adduction, internal rotation, external rotation; pain at 45^o^ of flexion and 0^o^ of internal and external rotation | MRI |
|  | Ragab et al. (2008) | Bone marrow oedema syndromes | Antalgic gait; pain on internal rotation; pain on external rotation | MRI |
|  | Saw et al. (2004) | Acetabular labral tear | Impingement test | Arthroscopy |
|  | Scott et al. (1999) | Femoral neck stress fracture | Antalgic gait; Patrick’s test | Radiography |
|  | Sherman et al. (2009) | Periprosthetic fracture of the femoral stem | Leg length (shortened limb) | Radiography |
|  | Siebenrock et al. (2003) | Femoroacetabular impingement associated with acetabular retroversion | Anterior impingement test | Radiography; MRI |
|  | Siebenrock et al. (2004) | Anterior femoroacetabular impingement, acetabular rim lesions with labral degeneration or partial labral tear | Restricted internal rotation; impingement test (FADIR) | MRI, Intraoperative confirmation |
|  | Sierra et al. (2009) | Femoroacetabular impingement and associated acetabular chondral damage | Impingement sign | MRA; surgery |
|  | Stahelin et al. (2008) | Cam-type femoroacetabular impingement and labral tears | Impingement sign | MRI; arthroscopy |
|  | Suenaga et al. (2002) | Labral tear in patient with dysplastic hips | Maximum flexion and internal rotation test; maximum flexion and external rotation test | Arthroscopy |
|  | Thorup et al. (2009) | Congenitally dislocated hip | Trendelenburg sign | Diagnosis part of inclusion criteria |
|  | Ueo et al. (1990) | Torn acetabular labrum | Straight leg-raising; pain on passive flexion-internal rotation; posterior push of the thigh that was flexed and adducted; Trendelenburg sign | Arthroscopy |
|  | Vann et al. (2007) | Protrusio acetabuli | Antalgic gait | Radiography |
|  | Vernon et al. (1997) | Transient osteoporosis of the hip | Pain on hip abduction | MRI |
|  | Voos et al. (2009) | Full thickness and high-grade partial-thickness tears of the gluteus medius muscle | Pain on flexion and internal rotation | Endoscopy |
|  | Wang et al. (2011) | Unilateral acetabular labral tears | FADIR test, FABER test, McCarthy test | MRA, Arthroscopy |
|  | Wisniewski et al. (2006) | Bilateral cam-type femoroacetabular impingement | Straight leg-raising; reverse straight leg raise; impingement test; Stinchfield test; FABER test | Radiography |

**References**:

1. Adkins MC, Sundaram M: **Radiologic case study. Insufficiency fracture of the acetabular roof in Paget's disease.** *Orthopedics* 2001, **24:**945, 1019-1020.

2. Amstutz HC, Mai LL, Schmidt I: **Results of Interlocking Wire Trochanteric Reattachment and Technique Refinements to Prevent Complications Following Total Hip Arthroplasty.** *Clinical Orthopaedics and Related Research* 1984, **183:**82-89.

3. Amstutz HC, Maki S: **Complications of trochanteric osteotomy in total hip replacement.** *J Bone Joint Surg Am* 1978, **60:**214-216.

4. Atilla B, Caglar O, Akgun RC: **Acute fracture of the acetabulum secondary to a convulsive seizure 3 years after total hip arthroplasty.** *Orthopedics* 2008, **31:**283.

5. Austin AB, Souza RB, Meyer JL, Powers CM: **Identification of abnormal hip motion associated with acetabular labral pathology.** *Journal of Orthopaedic & Sports Physical Therapy* 2008, **38:**558-565.

6. Beaulé PE, Zaragoza E, Motamedi K, Copelan N, Dorey FJ: **Three-dimensional computed tomography of the hip in the assessment of femoroacetabular impingement.** *Journal of orthopaedic research* 2005, **23:**1286-1292.

7. Beck M, Leunig M, Parvizi J, Boutier V, Wyss D, Ganz R: **Anterior femoroacetabular impingement: part II. Midterm results of surgical treatment.** *Clinical Orthopaedics and Related Research* 2004, **418:**67-73.

8. Bewyer D, Chen J: **Gluteus medius tendon rupture as a source for back, buttock and leg pain: Case report.** *The Iowa orthopaedic journal* 2005, **25:**187.

9. Bhinda H, Sarkar S: **Dissociation of the S-ROM^* metal-backed polyethylene acetabular liner. A case report and literature review.** *Acta orthopaedica belgica* 2003, **69:**86-88.

10. Binningsley D: **Tear of the acetabular labrum in an elite athlete.** *British Journal of Sports Medicine* 2003, **37:**84-88.

11. Bizzini M, Notzli HP, Maffiuletti NA: **Femoroacetabular Impingement in Professional Ice Hockey Players A Case Series of 5 Athletes After Open Surgical Decompression of the Hip.** *The American journal of sports medicine* 2007, **35:**1955-1959.

12. Bohannon Mason J: **Acetabular Labral Tears In The Athelete.** *Clinics in Sports Medicine* 2001, **20:**779-790.

13. Botti TP, Gent J, Martell JM, Manning DW: **Trunion fracture of a fully porous-coated femoral stem: case report.** *The Journal of arthroplasty* 2005, **20:**943-945.

14. Braun-Moscovici Y, Schapira D, Nahir AM: **Calcific Tendinitis of the Rectus Femoris.** *JCR: Journal of Clinical Rheumatology* 2006, **12:**298-300 210.1097/1001.rhu.0000249896.0000243792.0000249862.

15. Browder CDA, Erhard RE: **Decision making for a painful hip: a case requiring referral.** *The Journal of orthopaedic and sports physical therapy* 2005, **35:**738-744.

16. Brunner A, Horisberger M, Herzog RF: **Evaluation of a computed tomography–based navigation system prototype for hip arthroscopy in the treatment of femoroacetabular cam impingement.** *Arthroscopy: The Journal of Arthroscopic & Related Surgery* 2009, **25:**382-391.

17. Burnett RSJ, Della Rocca GJ, Prather H, Curry M, Maloney WJ, Clohisy JC: **Clinical presentation of patients with tears of the acetabular labrum.** *The Journal of Bone & Joint Surgery* 2006, **88:**1448-1457.

18. Byrd J: **Labral lesions: an elusive source of hip pain case reports and literature review.** *Arthroscopy: The Journal of Arthroscopic & Related Surgery* 1996, **12:**603-612.

19. Chan Y-S, Lien L-C, Hsu H-L, Wan Y-L, Lee MS, Hsu K-Y, Shih C-H: **Evaluating hip labral tears using magnetic resonance arthrography: a prospective study comparing hip arthroscopy and magnetic resonance arthrography diagnosis.** *Arthroscopy-the Journal of Arthroscopic and Related Surgery* 2005, **21:**1250e1251-1258.

20. Chen CK, Yeh L, Chang W-N, Pan H-B, Yang C-F: **MRI diagnosis of contracture of the gluteus maximus muscle.** *American Journal of Roentgenology* 2006, **187:**W169-W174.

21. Cibere J, Thorne A, Bellamy N, Greidanus N, Chalmers A, Mahomed N, Shojania K, Kopec J, Esdaile JM: **Reliability of the hip examination in osteoarthritis: Effect of standardization.** *Arthritis Care & Research* 2008, **59:**373-381.

22. Clohisy JC, Knaus ER, Hunt DM, Lesher JM, Harris-Hayes M, Prather H: **Clinical presentation of patients with symptomatic anterior hip impingement.** *Clin Orthop Relat Res* 2009, **467:**638-644.

23. Crevenna R, Keilani M, Wiesinger G, Nicolakis P, Quittan M, Fialka-Moser V: **Calcific trochanteric bursitis: resolution of calcifications and clinical remission with non-invasive treatment. A case report.** *Wiener klinische Wochenschrift* 2002, **114:**345.

24. Diez F: **Chiropractic management of patients with bilateral congenital hip dislocation with chronic low back and leg pain.** *Journal of manipulative and physiological therapeutics* 2004, **27:**280e281-288.

25. Eijer H, Leunig M, Mahomed M, Ganz R: **Cross-table lateral radiograph for screening of anterior femoral head-neck offset in patients with femoro-acetabular impingement.** *Hip Int* 2011, **11:**37–41.

26. Eijer H, Myers SR, Ganz R: **Anterior femoroacetabular impingement after femoral neck fractures.** *Journal of orthopaedic trauma* 2001, **15:**475-481.

27. Erhard RE, Egloff BP: **Patient with metastatic adenocarcinoma imitating lumbar herniated nucleus pulposis.[Erratum appears in J Manipulative Physiol Ther. 2005 May;28(4):290].** *J Manipulative Physiol Ther* 2004, **27:**569-573.

28. File P, Wood JP, Kreplick LW: **Diagnosis of hip fracture by the auscultatory percussion technique.** *The American journal of emergency medicine* 1998, **16:**173-176.

29. Fisher DA, Almand JD, Watts MR: **Operative Repair of Bilateral Spontaneous Gluteus Medius and Minimus Tendon RupturesA Case Report.** *The Journal of Bone & Joint Surgery* 2007, **89:**1103-1107.

30. Fitzgerald Jr RH: **Acetabular labrum tears: diagnosis and treatment.** *Clinical Orthopaedics and Related Research* 1995, **311:**60-68.

31. Fonstad P, Hooper RA: **Hip labral tears as a co-morbidity of low back and pelvic girdle pain following motor vehicle collisions: A case series.** *Journal of Back and Musculoskeletal Rehabilitation* 2008, **21:**245-251.

32. Fraitzl C, Käfer W, Nelitz M, Reichel H: **Radiological evidence of femoroacetabular impingement in mild slipped capital femoral epiphysis A MEAN FOLLOW-UP OF 14.4 YEARS AFTER PINNING IN SITU.** *Journal of Bone & Joint Surgery, British Volume* 2007, **89:**1592-1596.

33. Gédouin J-E, Duperron D, Langlais F, Thomazeau H: **Update to femoroacetabular impingement arthroscopic management.** *Orthopaedics & Traumatology: Surgery & Research* 2010, **96:**222-227.

34. Gille J, Krueger S, Aberle J, Boehm S, Ince A, Loehr JF: **Synovial chondromatosis of the hip: a case report and clinicopathologic study.** *Acta Orthop Belg* 2004, **70:**182-188.

35. Guanche CA, Sikka RS: **Acetabular labral tears with underlying chondromalacia: a possible association with high-level running.** *Arthroscopy: The Journal of Arthroscopic & Related Surgery* 2005, **21:**580-585.

36. Guis-Sabatier S, Pieri-Balandraud N, Garnier-Soumet P, Coste J, Roux H, Mattei J-P: **Pubic pain in athletes: a case due to an abscess in the obturator muscle.** *Revue du rhumatisme (English ed)* 1999, **66:**58.

37. Gurney B, Boissonnault WG, Andrews R: **Differential diagnosis of a femoral neck/head stress fracture.** *J Orthop Sports Phys Ther* 2006, **36:**80-88.

38. Hartmann A, Gunther KP: **Arthroscopically assisted anterior decompression for femoroacetabular impingement: technique and early clinical results.** *Arch Orthop Trauma Surg* 2009, **129:**1001-1009.

39. Hase T, Ueo T: **Acetabular labral tear: arthroscopic diagnosis and treatment.** *Arthroscopy* 1999, **15:**138-141.

40. Ichikawa J, Amano R, Haro H, Sato E, Koyama K, Hamada Y: **Fatigue fracture of the bilateral femoral neck in the elderly.** *Orthopedics* 2008, **31:**1141.

41. Ito K, Leunig M, Ganz R: **Histopathologic features of the acetabular labrum in femoroacetabular impingement.** *Clinical Orthopaedics and Related Research* 2004, **429:**262-271.

42. Ito K, Minka-II M-A, Leunig M, Werlen S, Ganz R: **Femoroacetabular impingement and the cam-effect.** *Journal of Bone & Joint Surgery, British Volume* 2001, **83:**171-176.

43. James S, Connell D, O'Donnell P, Saifuddin A: **Femoroacetabular impingement: bone marrow oedema associated with fibrocystic change of the femoral head and neck junction.** *Clinical radiology* 2007, **62:**472-478.

44. Kagan A, 2nd: **Rotator cuff tears of the hip.** *Clin Orthop Relat Res* 1999**:**135-140.

45. Kandemir U, Bharam S, Philippon MJ, Fu FH: **Endoscopic treatment of calcific tendinitis of gluteus medius and minimus.** *Arthroscopy: the journal of arthroscopic & related surgery: official publication of the Arthroscopy Association of North America and the International Arthroscopy Association* 2003, **19:**E4.

46. Keeney JA, Peelle MW, Jackson J, Rubin D, Maloney WJ, Clohisy JC: **Magnetic resonance arthrography versus arthroscopy in the evaluation of articular hip pathology.** *Clinical Orthopaedics and Related Research* 2004, **429:**163-169.

47. King R, Laugharne D, Kerslake R, Holdsworth B: **Case report primary obturator pyomyositis: A diagnostic challenge.** *Journal of Bone & Joint Surgery, British Volume* 2003, **85:**895-898.

48. Klässbo M, Harms-Ringdahl K, Larsson G: **Examination of passive ROM and capsular patterns in the hip.** *Physiotherapy Research International* 2003, **8:**1-12.

49. LaBan MM, Weir SK, Taylor RS: **'Bald Trochanter'Spontaneous Rupture of the Conjoined Tendons of the Gluteus Medius and Minimus Presenting as a Trochanteric Bursitis.** *American journal of physical medicine & rehabilitation* 2004, **83:**806-809.

50. Larson CM, Giveans MR: **Arthroscopic management of femoroacetabular impingement: early outcomes measures.** *Arthroscopy: The Journal of Arthroscopic & Related Surgery* 2008, **24:**540-546.

51. Lasanianos N, Kanakaris N, Giannoudis PV: **An occult acetabular fracture preceding a femoral neck fracture.** *Orthopedics* 2009, **32:**609.

52. Lavigne M, Boddu Siva Rama KR, Roy A, Vendittoli P-A: **Painful impingement of the hip joint after total hip resurfacing: a report of two cases.** *The Journal of arthroplasty* 2008, **23:**1074-1079.

53. Lequesne M, Djian P, Vuillemin V, Mathieu P: **Prospective study of refractory greater trochanter pain syndrome. MRI findings of gluteal tendon tears seen at surgery. Clinical and MRI results of tendon repair.** *Joint Bone Spine* 2008, **75:**458-464.

54. Liu K-Y, Wang S-J, Lin L-C: **Primary iliac muscle abscess due to Staphylococcus aureus.** *Journal of the Formosan Medical Association= Taiwan yi zhi* 1999, **98:**452.

55. Londers J, Van Melkebeek J: **Hip arthroscopy: outcome and patient satisfaction after 5 to 10 years.** *Acta Orthopædica Belgica* 2007, **73:**478.

56. Malas FÜ, Kara M, Kerimoglu Ü, Özçakar L: **An underestimated culprit of groin pain: Acetabular labrum tear.** *American journal of physical medicine & rehabilitation* 2007, **86:**690.

57. Matsuda DK: **Acute iatrogenic dislocation following hip impingement arthroscopic surgery.** *Arthroscopy: The Journal of Arthroscopic & Related Surgery* 2009, **25:**400-404.

58. McCarthy J, Puri L, Barsoum W, Lee J-a, Laker M, Cooke P: **Articular cartilage changes in avascular necrosis: an arthroscopic evaluation.** *Clinical Orthopaedics and Related Research* 2003, **406:**64-70.

59. McCarthy JC, Busconi B: **The role of hip arthroscopy in the diagnosis and treatment of hip disease.** *Orthopedics* 1995, **18:**753.

60. McMichael JC, Moed BR: **Failure of intertrochanteric fixation using a locking plate: a case report.** *Current Orthopaedic Practice* 2009, **20:**101-104.

61. Miozzari HH, Dora C, Clark JM, Nötzli HP: **Late repair of abductor avulsion after the transgluteal approach for hip arthroplasty.** *The Journal of arthroplasty* 2010, **25:**450-457. e451.

62. Mitchell B, McCrory P, Brukner P, O'Donnell J, Colson E, Howells R: **Hip joint pathology: clinical presentation and correlation between magnetic resonance arthrography, ultrasound, and arthroscopic findings in 25 consecutive cases.** *Clinical Journal of Sport Medicine* 2003, **13:**152-156.

63. Nötzli H, Wyss T, Stoecklin C, Schmid M, Treiber K, Hodler J: **The contour of the femoral head-neck junction as a predictor for the risk of anterior impingement.** *Journal of Bone & Joint Surgery, British Volume* 2002, **84:**556-560.

64. Nunley RM, Prather H, Hunt D, Schoenecker PL, Clohisy JC: **Clinical presentation of symptomatic acetabular dysplasia in skeletally mature patients.** *Journal of Bone & Joint Surgery, American Volume* 2011, **93:**17-21.

65. Padhy D, Park S-W, Jeong W-K, Lee D-H, Park JH, Han S-B: **Femoroacetabular Impingement Due to Synovial Chondromatosis of the Hip Joint.** *Orthopedics* 2009, **32:**921-923.

66. Philippon MJ, Maxwell RB, Johnston TL, Schenker M, Briggs KK: **Clinical presentation of femoroacetabular impingement.** *Knee Surgery, Sports Traumatology, Arthroscopy* 2007, **15:**1041-1047.

67. Pozzi G, Stradiotti P, Parra CG, Zagra L, Sironi S, Zerbi A: **Femoro-acetabular impingement: can indirect MR arthrography be considered a valid method to detect endoarticular damage? A preliminary study.** *Hip international: the journal of clinical and experimental research on hip pathology and therapy* 2009, **19:**386.

68. Quarrier NF, Wightman AB: **A ballet dancer with chronic hip pain due to a lesser trochanter bony avulsion: the challenge of a differential diagnosis.** *The Journal of orthopaedic and sports physical therapy* 1998, **28:**168.

69. Quintos-Macasa AM, Serebro L, Menon Y: **Transient synovitis of the hip in an adult.** *Southern medical journal* 2006, **99:**184-185.

70. Ragab Y, Emad Y, Abou-Zeid A: **Bone marrow edema syndromes of the hip: MRI features in different hip disorders.** *Clinical rheumatology* 2008, **27:**475-482.

71. Saw T, Villar R: **Footballer’s hip a report of six cases.** *Journal of Bone & Joint Surgery, British Volume* 2004, **86:**655-658.

72. Scott MP, Finnoff JT, Davis BA: **Femoral neck stress fracture presenting as gluteal pain in a marathon runner: case report.** *Archives of physical medicine and rehabilitation* 1999, **80:**236-238.

73. Sherman RA, Damron TA: **Penetration of a metallic femoral head through the acetabular shell.** *The Journal of arthroplasty* 2009, **24:**1143. e1147-1143. e1110.

74. Siebenrock K, Schoeniger R, Ganz R: **Anterior Femoro-Acetabular Impingement Due to Acetabular Retroversion Treatment with Periacetabular Osteotomy.** *The Journal of Bone & Joint Surgery* 2003, **85:**278-286.

75. Siebenrock KA, Wahab KH, Werlen S, Kalhor M, Leunig M, Ganz R: **Abnormal extension of the femoral head epiphysis as a cause of cam impingement.** *Clin Orthop Relat Res* 2004**:**54-60.

76. Sierra RJ, Trousdale RT: **Labral reconstruction using the ligamentum teres capitis: report of a new technique.** *Clinical Orthopaedics and Related Research* 2009, **467:**753-759.

77. Stähelin L, Stähelin T, Jolles BM, Herzog RF: **Arthroscopic offset restoration in femoroacetabular cam impingement: accuracy and early clinical outcome.** *Arthroscopy: The Journal of Arthroscopic & Related Surgery* 2008, **24:**51. e51-51. e58.

78. Suenaga E, Noguchi Y, Jingushi S, Shuto T, Nakashima Y, Miyanishi K, Iwamoto Y: **Relationship between the maximum flexion-internal rotation test and the torn acetabular labrum of a dysplastic hip.** *Journal of orthopaedic science* 2002, **7:**26-32.

79. Thorup B, Mechlenburg I, Søballe K: **Total hip replacement in the congenitally dislocated hip using the Paavilainen technique: 19 hips followed for 1.5–10 years.** *Acta orthopaedica* 2009, **80:**259-262.

80. Ueo T, Suzuki S, Iwasaki R, Yosikawa J: **Rupture of the labra acetabularis as a cause of hip pain detected arthroscopically, and partial limbectomy for successful pain relief.** *Arthroscopy: The Journal of Arthroscopic & Related Surgery* 1990, **6:**48-51.

81. Vann M, Onyike A, Gentry S, Banks W: **Atraumatic acetabular fracture in secondary protrusio acetabuli: a case report.** *Journal of surgical orthopaedic advances* 2007, **16:**93.

82. Vernon LF, Dooley JC, Neidorf DL: **Transient osteoporosis of the hip.** *Journal of clinical rheumatology: practical reports on rheumatic & musculoskeletal diseases* 1997, **3:**176.

83. Voos JE, Shindle MK, Pruett A, Asnis PD, Kelly BT: **Endoscopic repair of gluteus medius tendon tears of the hip.** *The American journal of sports medicine* 2009, **37:**743-747.

84. Wang WG, Yue DB, Zhang NF, Hong W, Li ZR: **Clinical diagnosis and arthroscopic treatment of acetabular labral tears.** *Orthop Surg* 2011, **3:**28-34.

85. Wisniewski SJ, Grogg B: **Femoroacetabular impingement: an overlooked cause of hip pain.** *American journal of physical medicine & rehabilitation* 2006, **85:**546-549.
